# Optimization Scheme for Hybrid RIS-Assisted ISAC System for Controllable Communication and Sensing

**DOI:** 10.3390/s26165303

**Published:** 2026-08-21

**Authors:** Zhishuo Deng, Bo Li, Hehang Wang

**Affiliations:** School of Electronics and Information Engineering, Liaoning University of Technology, Jinzhou 121001, China; 19560738107@163.com (Z.D.); whh13391983275@163.com (H.W.)

**Keywords:** integrated sensing and communication, hybrid reconfigurable intelligent surface, communication-sensing controllable coefficient, multiple-input multiple-output, Riemannian Hessian-based method, precoding matrix

## Abstract

Integrated sensing and communication (ISAC) systems are envisioned as a key enabler for next-generation wireless networks. To simultaneously achieve multi-function of communication and sensing for this system, a hybrid reconfigurable intelligent surface (RIS) comprising both active and passive reflecting elements is proposed. An index-wise importance score matrix and a factorized representation of complex reflection coefficients are optimized by introducing a communication-sensing controllable coefficient. A novel ISAC system is investigated, which exploits the low-power advantage of passive reflecting elements while retaining the signal amplification capability of active reflecting elements. In the multiple-input multiple-output (MIMO) communication networks, the RIS reflection coefficient matrix is adaptively optimized via a Riemannian Hessian-based method. At the same time, the transmit precoding matrix is obtained using an extended weighted minimum mean square error (WMMSE) and Lagrange multiplier methods. Compared with baseline schemes including active-only RIS, passive-only RIS, random-phase RIS, and non-RIS, the proposed scheme enables multi-mode adjustability of communication and sensing which can be easily transplanted in current ISAC system. Under the constraint of transmit power, it exhibits more robust performance on communication-sensing with varying number of RIS elements and different signal-to-noise ratios (SNRs).

## 1. Introduction

With the advantages of current wireless communication networks, the integrated sensing and communication (ISAC) system has become an important enabler for future applications. ISAC achieves mutual benefits between communication and sensing through shared spectrum, integrated hardware platforms, and joint signal processing [1,2,3,4,5]. The reconfigurable intelligent surface (RIS) features high energy efficiency and programmable beamforming capabilities. The unique characteristics of RIS provide abundant spatial degrees of freedom to proactively reshape the wireless propagation environment and suppress interference for communication rate or beam sensing [6,7].

Existing RIS-assisted ISAC designs balance communication and sensing objectives under transmit power, unit modulus, and quality-of-service (QoS) constraints. For example, Zhu et al. [8] optimized base station (BS) precoding, receive filtering, and RIS phases to enhance sum-rate while meeting a certain signal-to-interference-noise ratio (SINR). Luo et al. [9] proposed a joint beamforming framework with alternating optimization (AO) convergence. Further, Luo et al. [10] formulated beam and reflection with a robust solution strategy. Sankar et al. [11] treated RIS cases by accounting for user SINR and target illumination power. Typically, Shtaiwi et al. [6] adopted manifold optimization for communication rate under explicit constraints. On the other hand, Xu et al. [7] developed semidefinite relaxation (SDR) and one-dimensional iterative (ODI) methods. Zhang et al. [12] designed joint waveform and phase shift driven by mutual information (MI). Wang et al. [13] enforces a Cramer–Rao-bound (CRB) constraint in waveform and phase design, while Liu et al. [14] used signal-to-noise ratio (SNR) dual constraints to coordinate reliability. To reduce training overhead, Qian et al. [15] proposed a sensing-driven two-stage protocol that improves localization with near-lossless communications. Further, Chen et al. [16] introduced beam training and target sensing by exploiting angle-delay and Doppler-delay energy aggregation.

With respect to the cascaded attenuation in passive-only paths, the active-only RIS has been used to compensate path loss and strengthen sensing. Then, Yu et al. [17] improved radar SINR under desirable QoS via maximization-minimization (MM) and SDR and provided scaling laws with many reflecting elements. Liu et al. [18] employed block coordinate descent to jointly design the transmit beamformer, receive beamforming and active reflection coefficients. Zhu et al. [19] minimized CRB subject to SINR, revealing an amplification-noise trade-off. For simultaneous transmission and reflection by RIS (STAR-RIS), which extends coverage to full space with additional variables, Liu et al. [20] investigated active and passive beams under imperfect channel state information (CSI) using the AO, S-procedure, and SDR. Saikia et al. [21] compared STAR-RIS with passive-only RIS in ISAC system. Wang et al. [22] introduced Jain’s fairness index in STAR-RIS and non-orthogonal multiple access (NOMA) for fairness-rate co-optimization. Wei et al. [23] targeted secure NOMA-ISAC system for communication rate. Subsequently, Wang et al. [24] proposed a NOMA-assisted full-space STAR-RIS ISAC system to maximize the worst-case beam. For backscatter-RIS and system extensions, Wang et al. [25] integrated RIS and backscatter for an energy-efficient ISAC system. To raise sum-rate and sensing power, Nassar et al. [26] combined backscatter and NOMA with dynamic user clustering. Guo et al. [27] studied an RIS-assisted full-duplex uplink ISAC system via MM and penalty dual decomposition.

Yang et al. [28] investigated an RIS-assisted cooperative multi-element ISAC system to minimize power while guaranteeing rate and MI. By designing a joint track and resource, Wu et al. [29] addressed an RIS-unmanned aerial ISAC system. On access and security, Zuo et al. [30] integrated NOMA with the ISAC system and jointly optimized active/passive beams and power allocation. Chen et al. [31] designed a rate-splitting multiple access ISAC system to enhance SNR via joint rate-splitting and beamforming. Note that the communication rate is often achieved through artificial noise (AN) with transmission design [32]. Regarding learning paradigms, Liu et al. [33] employed deep reinforcement learning to optimize rate online with AN. Ye et al. [34] proposed unsupervised ISAC beam networks to approach near-optimality without labels.

Further, in current RIS structures, an active reflection unit refers to a unit capable of signal amplification. It typically integrates active circuits such as power amplifiers, which enable both phase modulation of the incident signal and amplitude amplification [35]. This helps overcome multi-path loss and random noise, which enhances both communication distance and signal strength for sensing applications [33]. Usually, the passive reflection element, which consists entirely of passive components and lacks signal amplification capability, can only adjust the phase of the incident signal. Such phase control enables low-power beam steering and beamforming, which facilitate intelligent reconfiguration of the wireless propagation environment [36]. It has been shown that beam alignment in sidelink-enabled vehicular networks can continuously provide angular measurements for both communication and sensing [37]. This synergy has profound significance for subsequent research on beam management.

Despite the significant progress achieved by the aforementioned studies, several challenges remain to be addressed. Firstly, the passive-only RIS is constrained by reflection gain; the active-only RIS introduces noise accumulation and power-budget coupling, leading to a conflict between communication and sensing. Secondly, multi-target scenarios subject to power and QoS constraints lead to highly non-convex optimization problems that suffer from slow convergence, sub-optimality, and constraint violations. Finally, under power-limited or low-to-middle SNR and multi-user/multi-target scenarios, realizing tunable and robust communication and sensing remains the key challenge in algorithm design. To address these issues, this study proposes a novel hybrid RIS for an ISAC system with randomly arranged active and passive reflecting elements. The unified mask-selection and layered complex-reflection modeling are explored. Under the transmit power constraint, a joint optimization problem is formulated to maximize a weighted sum of the communication rate and sensing beam gain, in which the BS precoding matrix and RIS phase shifts are jointly optimized to achieve an explicit and controllable communication–sensing trade-off. The main contributions of this study are as follows:

(1) *Decoupling channel noise and enhancing reflection*

We adopt the weighted sum of communication rate and sensing beam gain as the optimization objective, enabling the ISAC system to intelligently balance communication and sensing based on top-level requirements. As a result, it lays the foundation for mode adaptability. Joint optimization of RIS reflection coefficients and BS transmit precoding matrix effectively concentrates the signal energy toward intended directions, maximizing the overall system efficiency under power constraints.

(2) *Optimizing algorithms on BS and hybrid RIS alternately*

With fixed BS transmit precoding, the hybrid RIS reflection coefficients are optimized on a unit modulus and amplitude-constrained manifold via Riemannian gradients with the second-order information. With fixed RIS reflection coefficients, the BS transmit precoding is optimized based on the Lagrange multiplier method that balances communication rate and sensing beam gain. The optimized transmit precoding matrix is employed by the BS, and the optimized complex reflection coefficient matrix is sent to the RIS controller to adjust the state of each adjustable element.

(3) *Focusing signals and adjusting propagation directions*

The hybrid RIS structure employs a small number of active reflecting elements to achieve performance close to that of a fully active-only RIS while retaining the low-power advantages of a passive-only RIS. Passive reflecting elements are utilized to leverage their low-power beamforming capability, enabling flexible signal steering. Active reflecting elements provide signal amplification to mitigate path loss.

The remainder of this study is organized as follows: Section 2 designs the structure of the novel ISAC system, which includes the hybrid RIS structure and the MIMO model. Section 3 derives the optimization method for the proposed system that optimizes the RIS phase shift matrix, the transmit precoding matrix, and the sensing task. Section 4 evaluates the proposed scheme and compares it with existing methods in the literature. Finally, Section 5 concludes the study and discusses the future research directions.

**Notation**: Bold capital letters, bold lowercase letters, and lowercase letters respectively represent matrices, vectors, and scalars. ${\left[\cdot \right]}^{T}$ denotes the transpose operation, $E\left[\cdot \right]$ denotes the mathematical expectation, $\mathrm{diag}\left(\cdot \right)$ denotes the diagonal matrix with each element on the main diagonal. Furthermore, $\left|\cdot \right|$ denotes the modulus calculation of a complex number, $\left\|\cdot \right\|$ denotes the Euclidean norm, ${\left\|\cdot \right\|}_{2}$ denotes the *L*-two Euclidean norm, ${\left(\cdot \right)}^{*}$ denotes the complex conjugate vector, ≜ denotes the equality. $R\left(\cdot \right)$ denotes the real-part operator. $P_{\Phi }\left(\cdot \right)$ denotes projection operator onto the tangent space. $\exp\left(\cdot \right)$ denotes the exponential map.

## 2. Model Design on Proposed System

**Figure 1:**
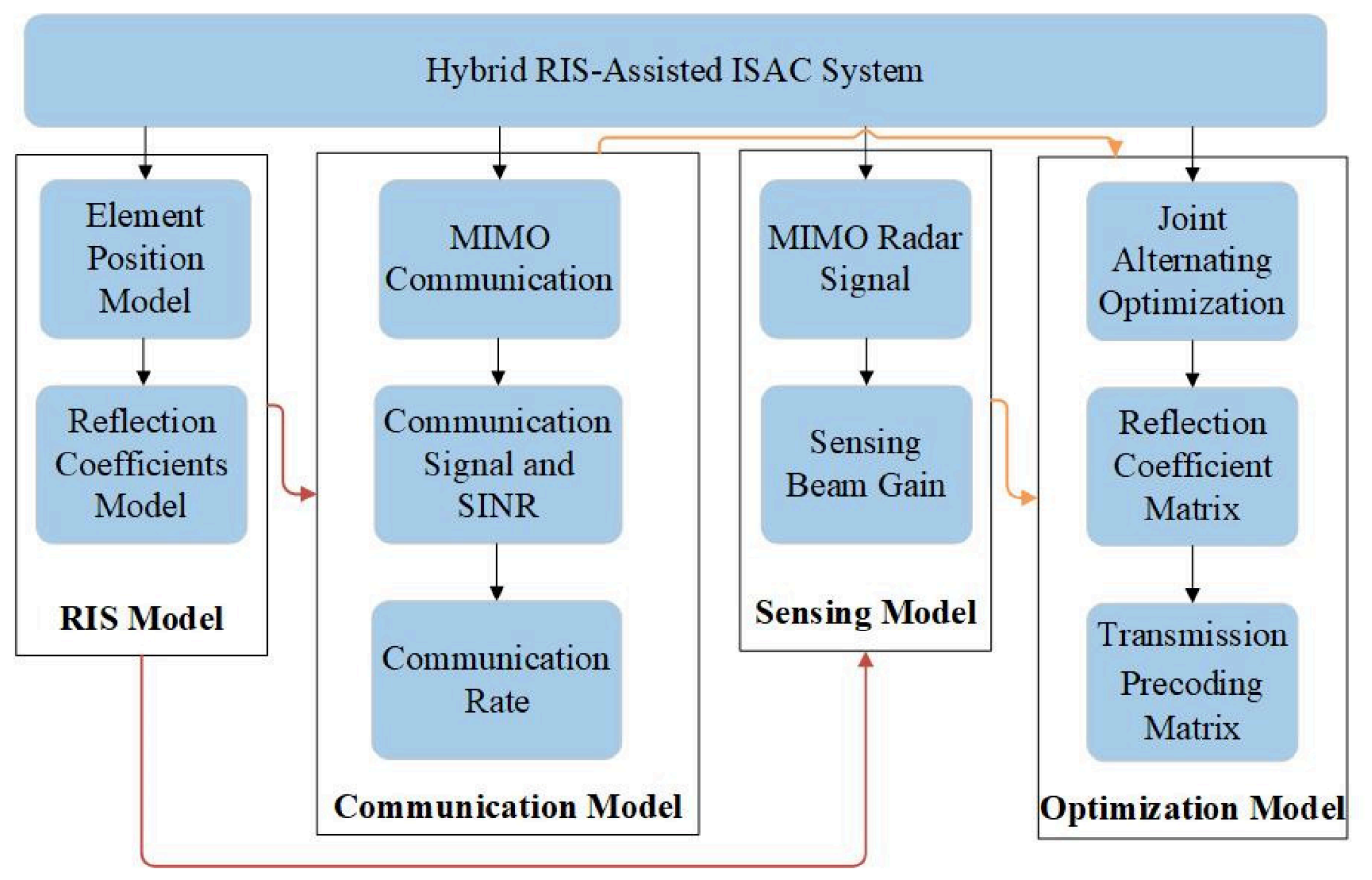
Proposed Framework of Communication and Sensing.

As shown in Figure 1, the hybrid RIS-assisted ISAC system provides a universal and comprehensive framework for current communication and sensing. The system mainly consists of the following functional components: The RIS model defines the RIS element positions and reflection coefficients, which serve as the basis for modeling MIMO radar signals. The communication model focuses on MIMO communication, which are evaluated communication performance by analyzing transmit SNR. The sensing model integrates the MIMO radar signal and beamforming for target detection or tracking. The optimization model, which employs the AO algorithm, jointly optimizes the communication and sensing objectives by updating the RIS phase shift matrix and the transmit precoding matrix. The communication capability is evaluated by the achievable rate.

**Figure 2:**
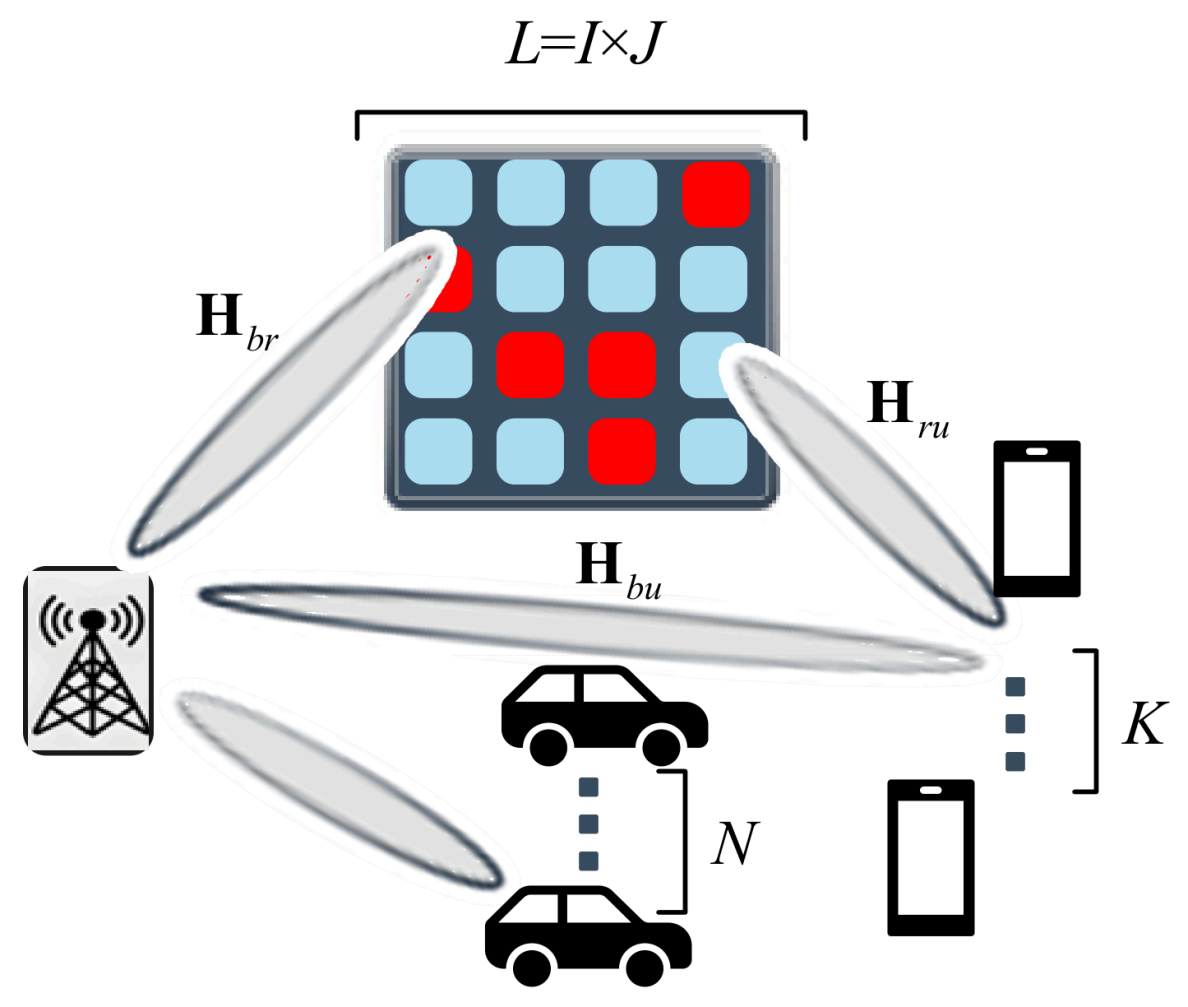
Hybrid RIS-Aided ISAC System Model.

In the system, the BS employs a uniform linear array (ULA) with M antennas and a spatially hybrid architecture, providing communication service to *K* single-antenna users while sensing N point targets. To focus on the proposed optimization framework, the BS is assumed to employ standard self-interference cancellation technology with ideal cancellation performance. The simple model is illustrated in Figure 2.

### 2.1. Model of RIS Element Position

Assume that the hybrid RIS consists of *L* (*L* = *I* × *J*) reflecting elements, where *I* and *J* represent the number of rows and columns of the RIS matrix, respectively. The importance score for the RIS element (*i*, *j*) is given by

(1) $$\delta_{i,j}=\left|h_{ru,i,j}\right|\cdot \left\|h_{br,i,j}\right\|$$

where $h_{ru,i,j}$ denotes the channel coefficient from RIS to user, and $h_{br,i,j}$ denotes the channel coefficient from BS to RIS.

Then, the importance score matrix ${\left[\delta_{i,j}\right]}_{I\times J}$ can be vectored in the column-major order to obtain the importance score vector $\delta ={\left[\delta_{1},\delta_{2},\ldots ,\delta_{L}\right]}^{T}\in {\mathbb{C}}^{L\times 1}$, which is sorted in descending order to obtain the priority index p as

(2) $$\delta_{p_{1}}\geq \delta_{p_{2}}\geq \cdots \geq \delta_{p_{L}}$$

where $p_{l}$ denotes the *l*th element, and $p_{1}$ corresponds to the position of the most important element. According to the obtained priority index p, the active/passive element indicator vector is $d_{p}={\left[d_{p_{1}},d_{p_{2}},\ldots ,d_{p_{L}}\right]}^{T}\in {\left\{0,1\right\}}^{L\times 1}$, where $d_{p_{l}}=1$ represents active reflecting elements, and $d_{p_{l}}=0$ represents passive reflecting elements. Note that by using a small number of active reflecting elements, the proposed system achieves a performance approaching that of a fully active RIS. It maintains the low-power advantage of a passive-only RIS. This avoids the deployment of expensive and high-power traditional sensing infrastructure and reduces the hardware overhead for implementing integrated sensing functions.

### 2.2. Model of RIS Complex Reflection

Define the gain amplitudes of each element of RIS as:

(3) $$\gamma_{l}=1+\left(\gamma -1\right)\cdot \frac{\delta_{p_{l}}-\delta_{\min}}{\delta_{\max}-\delta_{\min}}$$

where $\delta_{\min}$ and $\delta_{\max}$ denote the values obtained by vectorizing the principal diagonal of the importance scoring matrix, and then obtaining the minimum and maximum values; γ>1 is the amplitude gain of active reflecting elements.

Assume that the complex reflection coefficient at position *p_i_* in vector $d_{p}$ as:

(4) $$\phi_{p_{l}}=\left(1-d_{p_{l}}\right)e^{j\theta_{p_{l}}}+d_{p_{l}}\gamma_{p_{l}}e^{j\theta_{p_{l}}}$$

where $\theta_{p_{l}}$ denotes the phase shift, γ>1 denotes the amplitude gain of active reflecting elements.

For passive reflecting elements, the complex reflection coefficient as:

(5) $$\phi_{p_{l}}=e^{j\theta_{p_{l}}}$$

For active reflecting elements, the complex reflection coefficient as:

(6) $$\phi_{p_{l}}=\gamma e^{j\theta_{p_{l}}}$$

The complex reflection coefficient matrix for all RIS reflecting elements can be written as a diagonal matrix:

(7) $$\begin{array}{c} \Phi =\mathrm{diag}\left(\phi_{p_{1}},\phi_{p_{2}},\cdots ,\phi_{p_{L}}\right)\\ \;\;\;\;\;\;\;=\mathrm{diag}\left(e^{j\theta }\right)+\mathrm{diag}\left(\left(\gamma -1\right)d_{p}\right)\mathrm{diag}\left(e^{j\theta }\right) \end{array}$$

where $\theta ={\left[\theta_{1},\ldots ,\theta_{L}\right]}^{T}$ denotes the phase shift vector, with unit modulus phase $\mathrm{diag}\left(e^{j\theta }\right)$ applied to all elements. Thus, the complex reflection coefficient matrix Φ incorporates both the passive phase shift components and active gain components. For active reflecting elements, the additional gain is introduced, $\mathrm{diag}\left(\left(\gamma -1\right)d_{p}\right)$ can be captured further by the vector $d_{p}$.

**Remark 1.**  *The proposed hybrid RIS architecture is designed to overcome the inherent limitations of its fully active and fully passive counterparts in ISAC systems. On the randomly partitioned RIS array, a small fraction of active reflecting elements is selected for precise signal amplification to mitigate propagation attenuation. However, the vast majority of passive reflecting elements retain their low-power consumption and noise-free phase control capabilities. The proposed structure achieves a Pareto-optimal trade-off between beamforming gain and total noise suppression. On the other hand, the optimal active/passive allocation for all L reflecting elements is determined by Equation (1) based on the importance score matrix. The matrix encapsulates the cascaded channel conditions. The RIS controller receives the binary configuration vector and maps it to the bias control signals for each reflecting element. Each element is equipped with a PIN diode. By applying a forward bias, the PIN diode connects the element to the integrated low-noise amplification circuit that can operate in the active mode.*

### 2.3. Model of MIMO Communication System

Assume the transmit symbol vector of the users, denoted by $s={\left[s_{1},\ldots ,s_{K}\right]}^{T}\in {\mathbb{C}}^{K\times 1}$, follows a complex Gaussian distribution with zero mean and unit variance. The channel conditions remain constant within a coherence block consisting of time slots. The unit power of the transmit symbol is given by:

(8) $$E\left[{\left|s_{k}\right|}^{2}\right]=1$$

According to the principle of channel multipath propagation, the signal $y^{\mathrm{com}}\in {\mathbb{C}}^{K\times 1}$ received by *K* users contains both the direct channel from BS to user and the RIS-assisted reflection channel:

(9) $$y^{\mathrm{com}}=\left(H_{bu}+H_{ru}\Phi H_{br}\right)x+w=H_{b}x+w$$

where $H_{bu}\in {\mathbb{C}}^{K\times M}$ denotes the channel matrix from BS to user, $H_{br}\in {\mathbb{C}}^{L\times M}$ denotes the channel matrix from BS to RIS, $H_{ru}\in {\mathbb{C}}^{K\times L}$ denotes the channel matrix from RIS to user, $H_{b}\in {\mathbb{C}}^{K\times M}$ denotes the equivalent channel matrix combining direct channel with reflection channel, $\Phi \in {\mathbb{C}}^{L\times L}$ denotes the RIS reflection matrix, and $w\in {\mathbb{C}}^{K\times 1}$ denotes the additive white Gaussian noise (AWGN).

The transmit signal of user $x\in {\mathbb{C}}^{M\times 1}$ is defined as:

(10) x=Fs

where $F=\left[f_{1},\ldots ,f_{K}\right]\in {\mathbb{C}}^{M\times K}$ denotes the transmit precoding matrix, and $f_{k}\in {\mathbb{C}}^{M\times 1}$ is the transmit precoding vector for user *k*.

Then, the optimal receive equalizer for user *k* is given by:

(11) $$\begin{array}{l} u_{k}={\left(\sum_{q=1}^{K}{\left|h_{b,k}f_{q}\right|}^{2}+\sigma_{w}^{2}\right)}^{-1}h_{b,k}f_{k}\\ \;={\left(\sum_{q=1}^{K}h_{b,k}f_{q}f_{q}^{H}h_{b,k}^{H}+\sigma_{w}^{2}\right)}^{-1}h_{b,k}f_{k} \end{array}$$

where $\sigma_{w}^{2}=\sigma^{2}+\sum_{p_{i}}{\left|h_{ru,i,j}\gamma_{D_{p}}\sigma_{\mathrm{act}}\right|}^{2}$ denotes the power of noise [38], $h_{b,k}\in {\mathbb{C}}^{1\times M}$ denotes the channel that points to user *k* in the cascaded channel, and $\sigma_{\mathrm{act}}$ represents the thermal noise of active components. Specifically, the cascaded amplified noise and the environmental noise are merged into an equivalent aggregate noise. The receive equalizer $u_{k}$ is introduced as an auxiliary variable to formulate the mean square error (MSE) function, thereby facilitating the decoupling of the precoding design. Furthermore, although each user is equipped with only a single antenna, the received signal is still corrupted by multi-user interference.

The SINR of user *k* as:

(12) $$\gamma_{k}=\frac{{\left|h_{b,k}f_{k}\right|}^{2}}{\sum_{q=1,q\neq k}^{K}{\left|h_{b,k}f_{q}\right|}^{2}+\sigma_{w}^{2}}$$

The achievable sum rate R of the *K* users as:

(13) $$R=\overset{K}{\underset{k=1}{\sum }}\log_{2}\left(1+\gamma_{k}\right)$$

### 2.4. Model of MIMO Radar Signal

In this system, the user transmits symbols independently, and the covariance of the transmitted signal is given by:

(14) $$R_{s}=E\left[xx^{H}\right]=\sum_{k=1}^{K}f_{k}f_{k}^{H}$$

Suppose the BS simultaneously senses N target directions. Let the receive and transmit array vectors [39] be $a\left(\phi_{B,r}\right)\in {\mathbb{C}}^{M\times 1}$ and $a_{t}\left(\phi_{B,r}\right)\in {\mathbb{C}}^{M\times 1}$, target scattering coefficient is $\beta_{r}$. Then, the BS receives the sensing signal $y_{r}^{\mathrm{sens}}\in {\mathbb{C}}^{M\times 1}$ in the *r*th direction as:

(15) $$\begin{array}{l} y_{r}^{\mathrm{sens}}=\sum_{r=1}^{N}\beta_{r}a\left(\phi_{B,r}\right)a_{t}^{H}\left(\phi_{B,r}\right)x+\beta_{r}a\left(\phi_{B,r}\right)b^{H}\left(\phi_{R,r}\right)\Phi H_{br}x+n\\ \;\;\approx \beta_{r}a\left(\phi_{r}\right)\left[\underset{≜h_{\mathrm{rad}}^{H}\left(\phi_{r},\phi_{p_{L}}\right)}{\underset{︸}{a_{t}^{H}\left(\phi_{r}\right)+b^{H}\left(\phi_{r}\right)\Phi H_{br}}}\right]x+n \end{array}$$

where $b\left(\phi_{R,r}\right)\in {\mathbb{C}}^{L\times 1}$ is the directional vector of RIS, $n\in {\mathbb{C}}^{M\times 1}$ is the environmental noise received by the *M* antennas of the BS, with zero mean and covariance matrix $\sigma_{n}^{2}I_{M}$. To simplify the simulation setup and while maintaining the general assumption, the RIS is deployed at a location close to the BS. The target directions of the BS and RIS can be regarded as identical, which is $\phi_{B,r}\approx \phi_{R,r}≜\phi_{r}$.

The proposed system uses the unified transmit signal to detect the potential target state in a specified spatial direction by evaluating the echo power in direction *r*, while omitting the constant term:

(16) $$\begin{array}{l} E\left[{\left\|y_{r}^{\mathrm{sens}}\right\|}^{2}\right]\approx E\left[h_{\mathrm{rad}}^{H}\left(\phi_{r},\phi_{p_{L}}\right)xx^{H}h_{\mathrm{rad}}\left(\phi_{r},\phi_{p_{L}}\right)\right]\\ \;\;\;\;\;=\sum_{k=1}^{N}h_{\mathrm{rad}}^{H}f_{k}f_{k}^{H}h_{\mathrm{rad}} \end{array}$$

(17) $$E_{r}=\left[h_{\mathrm{rad}}\left(\phi_{r},\phi_{p_{L}}\right),\cdots ,h_{\mathrm{rad}}\left(\phi_{N},\phi_{p_{L}}\right)\right]=\left[e_{1},e_{2},\cdots ,e_{N}\right]$$

where $e_{r}$ is the sensing element related to the *r*th direction, and the $E_{r}$ represents the response matrix in N detection directions:

(18) $$P={\left\|\sum_{k=1}^{K}E_{r}f_{k}\right\|}^{2}=\mathrm{tr}\left(\sum_{k=1}^{K}E_{r}f_{k}f_{k}^{H}E_{r}^{H}\right)$$

Note that increasing the sensing beam gain P can directly improve the received echo signal strength, which enhances the target detection and estimation performance.

## 3. Method Optimization on Proposed System

Given the BS transmit power limitation, the proposed system jointly optimizes the transmit precoding matrix and the RIS phase shift matrix to maximize an achievable performance while satisfying the transmit power constraint. The optimization problem is formulated as:

(19) $$\begin{array}{l} \underset{\left\{F\right\},\left\{\Phi_{k}\right\}}{\max}\left(\frac{\alpha R}{R_{\max}}+\frac{\left(1-\alpha \right)P}{P_{\max}}\right)\\ =\underset{\left\{F\right\},\left\{\Phi_{k}\right\}}{\max}\left(\frac{\alpha }{R_{\max}}\overset{K}{\underset{k=1}{\sum }}\log_{2}\left(1+\gamma_{k}\right)+\frac{1-\alpha }{P_{\max}}\mathrm{tr}\left(\sum_{k=1}^{K}E_{r}f_{k}f_{k}^{H}E_{r}^{H}\right)\right)\\ \begin{array}{c} s.t.\;\begin{array}{cc} \sum_{k=1}^{K}{\left\|f_{k}\right\|}_{2}^{2}\leq P_{T}, & \theta_{l}\in \left[0,2\pi \right],\forall l=1,2,\cdots ,L \end{array} \end{array} \end{array}$$

where the communication-sensing controllable coefficient $\alpha \in \left[0,1\right]$ for communication and sensing is used to balance dual-mode functions. $R_{\max}$ is the maximum communication rate by setting α=1, the problem is simplified to a pure communication rate maximization problem, and its convergence target value is $R_{\max}$. Similarly, by setting α=0, the problem is simplified to a pure sensor gain maximization problem, and the convergence value is the maximum sensing gain $P_{\max}$, and $P_{T}$ is the maximum transmit power of BS. Both terms in Equation (19) are normalized, ensuring consistent value ranges in a dimensionless manner, which facilitates control of α. The proof of the conclusion in this section is presented in Appendix A.

### 3.1. Optimizing RIS Complex Reflection Coefficient Matrix

When the precoding matrix F is fixed, each element on the diagonal of Φ satisfies the unit modulus constraint:

(20) $$\begin{array}{l} \underset{\Phi }{\min}\;f\left(\Phi \right)\\ s.t.\;M=\{\Phi \in C^{L}:\left|\phi_{l}\right|=1,\forall l=1,2,\cdots ,L\} \end{array}$$

The objective function $f\left(\Phi \right)$ in the embedding space ${\mathbb{C}}^{L}$ relative to Φ is the Euclidean gradient:

(21) $$\nabla f\left(\Phi \right)=\left(\frac{\partial f}{\partial \phi_{1}},\frac{\partial f}{\partial \phi_{2}},\ldots ,\frac{\partial f}{\partial \phi_{L}}\right)$$

When optimizing on unit circle manifold, the Euclidean gradient is projected onto the tangent space of manifold at the current point:

(22) $$P_{\Phi }\left(\nabla f(\Phi \right)\;=\nabla f\left(\Phi \right)-R\left(\nabla f(\Phi )⊙\Phi^{H}\right)⊙\Phi$$

where $R\left(\cdot \right)$ is the real part of complex number.

Let the complex reflection matrix at the iteration *t* be denoted by $\Phi_{t}$. The update based on Riemannian gradient descent on complex circle manifold is given by:

(23) $$\Phi_{t+1\;}=\exp_{\Phi_{t}\;}\left(-\eta_{t}\;\nabla_{M}\;f\left(\Phi_{t}\right)\right)\;$$

To improve the manifold search efficiency, the proposed approach exploits second-order geometric information, which characterizes the local curvature and facilitates adaptive step-size adjustment. Usually, the passive reflecting elements are constrained by unit mode (non-convex) and the active reflecting elements are constrained by amplitude (convex). The Riemannian Hessian-based algorithm can adaptively optimize the manifold. By constraining both the non-convex unit modulus of the passive elements and the amplitude of active elements within the same geometric space, the optimization of all element phases is coordinated as a single block variable. Then, the Riemannian Hessian of the objective function on the complex circle manifold is given as:

(24) $$\mathrm{Hess}\left(\Phi_{t}\;\right)=\nabla_{M}^{2}f\left(\Phi_{t}\right)$$

At each iteration, the update direction is adjusted by Riemannian gradient and curvature-scaled step size. The update at the iteration *t* + 1 can be extended as:

(25) $$\Phi_{t+1\;}=\exp\;\left(-\eta_{t}\;{\mathrm{Hess}}^{-1}\left(\Phi_{t}\;\right)\;\;\nabla_{M}\;f\left(\Phi_{t}\right)\right)\;$$

where ${\mathrm{Hess}}^{-1}\left(\cdot \right)\;\;$ is the solution in Riemannian Newton direction, and ${\mathrm{Hess}}^{-1}\left(\Phi_{t}\;\right)\;\;\nabla_{M}\;f\left(\Phi_{t}\right)$ is the search direction determined using the curvature in advance.

Further, the adaptive step size mechanism for dynamic adjustment of local curvature is given by:

(26) $$\eta_{t}\;=\frac{\xi_{t}}{{\left\|\nabla_{M}^{2}f\left(\Phi_{t}\right)\right\|}^{2}+\zeta_{t}\left\|\mathrm{Hess}\left(\Phi_{t}\right)\right\|}$$

where the term ${\left\|\nabla_{M}^{2}f\left(\Phi_{t}\right)\right\|}^{2}$ is used to measure current gradient. When the gradient is large, the step size is reduced to avoid oscillations. The term $\left\|\mathrm{Hess}\left(\Phi_{t}\right)\right\|$ is used to measure the local curvature. When the curvature is large, the step size is reduced to optimize curvature. The hyperparameter ξ is used to control the overall step size scale with the threshold ε and maximum number of iterations $T_{\Phi }$:

(27) $$\;\xi_{t}=\xi_{0}\exp\left(-\frac{t}{T_{\Phi }}\varepsilon \right)$$

The hyperparameter ζ is used to balance the importance between gradient and curvature with the threshold ϵ:

(28) $$\;\zeta_{t}=\;\zeta_{0}\left(1+\frac{{\left\|\nabla_{M}^{2}f\left(\Phi_{t}\right)\right\|}^{2}}{{\left\|\nabla_{M}^{2}f\left(\Phi_{0}\right)\right\|}^{2}+\epsilon }\right)$$

Finally, the complex reflection coefficient matrix is achieved when the convergence condition satisfies the given maximum number of iterations. The detailed process of the above formula can be found in Appendix B.

**Remark 2.**  *The hybrid RIS reflection model incorporates both a universal phase-shift component common to all elements, and an active amplification gain specific to the active ones. The proposed reflection matrix formulation, which explicitly characterizes both the passive phase shifts and active amplification gains, facilitates the coordinated optimization of active and passive elements. Consequently, the structural model is incorporated into the subsequent joint optimization procedure, which reduces the overall computational overhead.*

### 3.2. Optimizing the Transmit Precoding Matrix

With a fixed RIS phase shift matrix, the precoding subproblem can be efficiently solved by leveraging the weighted minimum mean square error (WMMSE) framework. To accommodate the dual-functional nature of ISAC, we extend the conventional WMMSE by reformulating the non-convex rate maximization as an equivalent weighted MSE minimization problem, augmented with a sensing-induced quadratic regularization term to guarantee the sensing beam gain.

Equation (13) can be rewritten as:

(29) $$R=\overset{K}{\underset{k=1}{\sum }}\log_{2}\left(1+\gamma_{k}\right)=\underset{\left\{\omega_{k}\right\}}{\max}\left(\log_{2}\omega_{k}-\frac{\omega_{k}e_{k}-1}{\ln2}\right)$$

where the mean squared error $e_{k}$ is defined as:

(30) $$\begin{array}{l} e_{k}=E\left[{\left|{\hat{s}}_{k}-s_{k}\right|}^{2}\right]\\ \;={\left|u_{k}^{*}h_{b,k}f_{k}-1\right|}^{2}+\sum_{q\neq k}{\left|u_{k}^{*}h_{b,k}f_{q}\right|}^{2}+\sigma_{w}^{2}{\left|u_{k}^{*}\right|}^{2}\\ \;=\sum_{q=1}^{K}{\left|u_{k}^{*}h_{b,k}f_{q}\right|}^{2}+1-2R\left(u_{k}^{*}h_{b,k}f_{k}\right)+\sigma_{w}^{2}{\left|u_{k}^{*}\right|}^{2} \end{array}$$

The corresponding optimal weight as:

(31) $$\omega_{k}=\frac{1}{e_{k}^{\min}}$$

Then, Equation (19) can be rewritten as:

(32) $$\begin{array}{l} \underset{f_{k}}{\max}\left(\begin{array}{l} \frac{\alpha }{R_{\max}}\overset{K}{\underset{k=1}{\sum }}\left(\log_{2}\omega_{k}-\frac{1}{\ln2}\left(\overset{K}{\underset{k=1}{\sum }}\omega_{k}\left(\begin{array}{l} f_{q}^{H}h_{b,k}^{H}u_{k}u_{k}^{*}h_{b,k}f_{q}+1\\ -2R\left(u_{k}^{*}h_{b,k}f_{k}\right)+\sigma_{w}^{2}{\left|u_{k}^{*}\right|}^{2} \end{array}\right)\right)\right)\\ +\frac{1-\alpha }{P_{\max}}\mathrm{tr}\left(\sum_{k=1}^{K}E_{r}f_{k}f_{k}^{H}E_{r}^{H}\right) \end{array}\right)\\ \begin{array}{c} s.t.\begin{array}{cc} \sum_{k=1}^{K}{\left\|f_{k}\right\|}_{2}^{2}\leq P_{T}, & \theta_{l}\in \left[0,2\pi \right],\forall l=1,2,\cdots ,L \end{array} \end{array} \end{array}$$

For the sake of clarity, the objective function is defined as:


(33) $$\begin{array}{l} f\left(f_{k}\right)=\frac{\alpha }{R_{\max}}\overset{K}{\underset{k=1}{\sum }}\left(\log_{2}\omega_{k}-\frac{1}{\ln2}\left(\overset{K}{\underset{q=1}{\sum }}\omega_{q}\left(\begin{array}{l} f_{q}^{H}h_{b,k}^{H}u_{k}u_{k}^{*}h_{b,k}f_{q}+1\\ -2R\left(u_{k}^{*}h_{b,k}f_{k}\right)+\sigma_{w}^{2}{\left|u_{k}^{*}\right|}^{2} \end{array}\right)\right)\right)\\ \;\;+\frac{1-\alpha }{P_{\max}}\mathrm{tr}\left(\sum_{k=1}^{K}E_{r}f_{k}f_{k}^{H}E_{r}^{H}\right) \end{array}$$

With respect to the total transmit power constraint, the Lagrange multiplier χ is introduced, and the partial derivative of Equation (33) with respect to $f_{k}$ is computed as:

(34) $$\frac{\partial f\left(f_{k}\right)}{\partial f_{k}^{*}}=\frac{-\alpha }{R_{\max}\ln2}\overset{K}{\underset{q=1}{\sum }}\omega_{q}\left(h_{b,k}^{H}u_{q}u_{q}^{*}h_{b,q}f_{k}+h_{b,k}^{H}u_{k}\right)+\frac{1-\alpha }{P_{\max}}E_{r}^{H}E_{r}f_{k}+\chi f_{k}$$

Setting the result of Equation (34) to zero, it gives:

(35) $$\left(\underset{A}{\underset{︸}{\frac{1-\alpha }{P_{\max}}E_{r}^{H}E_{r}-\frac{\alpha }{R_{\max}\ln2}\overset{K}{\underset{q=1}{\sum }}\omega_{q}h_{b,k}^{H}u_{q}u_{q}^{*}h_{b,q}}}+\chi I\right)f_{k}=\underset{b_{k}}{\underset{︸}{\frac{\alpha \omega_{k}}{R_{\max}\ln2}h_{b,k}^{H}u_{k}}}$$

The matrix $A\in {\mathbb{C}}^{M\times M}$ in Equation (35), which eigenvalue decomposition is expressed as:

(36) $$A=Z\Lambda Z^{H}$$

where $\Lambda =\mathrm{diag}\left(\lambda_{1},\lambda_{2},\cdots ,\lambda_{M}\right)$ is the diagonal matrix $Z=\left[z_{1},z_{2},\cdots ,z_{M}\right]\in {\mathbb{C}}^{M\times M}$ is the eigenvalue matrix, $z_{m}\in {\mathbb{C}}^{M\times 1}$ is the unit-norm eigenvector matrix.

In order to reduce the complexity of matrix inversion, the Woodbury identity method is adopted. Thus, $f_{k}$ can be solved using Equation (35):

(37) $$\begin{array}{l} f_{k}={\left(A+\chi I\right)}^{-1}b_{k}\\ \;=\frac{1}{\chi }b_{k}-\frac{1}{\chi^{2}}Z{\left(\Lambda^{-1}+\frac{1}{\chi }Z^{H}Z\right)}^{-1}Z^{H}b_{k} \end{array}$$

After the above result is substituted into Equation (32), the actual transmit power related to χ as:

(38) $$P\left(\chi \right)=\sum_{k=1}^{K}{\left\|Z{\left(\Lambda +\chi I\right)}^{-1}\underset{c_{k}}{\underset{︸}{Z^{H}b_{k}}}\right\|}_{2}^{2}=\sum_{k=1}^{K}\sum_{m=1}^{M}\frac{{\left|c_{k,m}\right|}^{2}}{{\left(\lambda_{m}+\chi \right)}^{2}}\leq P_{T}$$

where $c_{k}$ is transformation from standard basis of $b_{k}$ to eigenbasis, and ${\left|c_{k,m}\right|}^{2}$ is the power gain for user *k* at antenna *m*.

Further, the derivative of Equation (32) as:

(39) $$\frac{\partial P\left(\chi \right)}{\partial \chi }=-2\sum_{k=1}^{K}\sum_{m=1}^{M}\frac{{\left|c_{k,m}\right|}^{2}}{{\left(\lambda_{m}+\chi \right)}^{3}}<0$$

The Newton update for χ at the iteration *t* + 1 as:

(40) $$\chi_{t+1}=\chi_{t}-\frac{P\left(\chi_{t}\right)-P_{T}}{P^{\prime }\left(\chi_{t}\right)}\geq 0$$

Finally, the optimization algorithm is summarized in Algorithm 1 (Pseudocode of Proposed Algorithm). Some details can be found in Appendix C.
**Algorithm 1.** Alternating Riemannian gradient & Lagrange-multiplier joint optimization**Input:** *M*, *K*, *N*, *L*, $H_{br}$, $H_{bu}$, $H_{ru}$, $R_{\max}$, $P_{\max}$, α, $\sigma_{w}^{2}$ **Output** optimized $\Phi_{t+1}$, F, and achieved R, P.1:$\mathrm{Compute}\;\delta_{i,j}$ using Equation (1);2:$\mathrm{Vectorize}\;{\left[\delta_{i,j}\right]}_{I\times J}$ in column-major order to get ${\left[\delta_{1},\delta_{2},\ldots ,\delta_{L}\right]}^{T};$
3:Compute priority index p satisfying Equation (2);4:Assign active and passive reflecting elements based on p;5:**Initialize** reflection coefficients;6:Compute complex reflection coefficient $\phi_{p_{i}}$ using Equation (4);7:**If** passive: using Equation (5); 8:**If** active: using Equation (6);9:**Initialize** precoding matrix $f_{0};$
10:**while**11:   %***Optimize*** $\Phi_{t+1}$ ***with fixed* F *using Riemannian* *Hessian algorithm***12: **for** inner iterations $t=1\;\mathrm{to}\;T_{\Phi };$
13:   Compute equivalent channel and $\gamma_{k}$ using Equation (12);14:   Compute communication rate R using Equation (13);15:   Compute sensing gain P using Equation (18);16:   Construct objective function using Equation (19);17:   Compute Euclidean gradient $\nabla f\left(\Phi \right)$ using Equation (21);18:   Compute Riemannian gradient using Equation (22);19:   Compute Riemannian Hessian function $\mathrm{Hess}\left(\Phi_{t}\;\right)$ using Equation (21); and compute adaptive step size $\eta_{t}$ using Equations (26)–(28);20:   Update $\Phi_{t+1\;}$ using Equation (25), extended from Equation (23).21:   %***End RIS optimization***22:   %***Optimize* F *with fixed*** $\Phi_{t}$ ***using Lagrange* *Newton method***23:Compute equivalent channel and $e_{k}$ using Equation (30), and compute optimal weight $\omega_{k}$ using Equation (31);24:Rewrite communication rate R using Equation (29), and construct objective using Equations (32) and (33);25: Introduce Lagrange multiplier χ for power constraint;26: **for** inner iterations *t* = 1 to *T***_F_**27:   Solve $\partial f\left(f_{k}\right)/\partial f_{k}^{*}$ using Equation (34) to get Equation (35);28:   Perform eigenvalue decomposition using Equation (36);29:   Solve $f_{k}$ using Equation (37);30:   Compute power $P\left(\chi \right)$ using Equation (38);31:   Compute differential using Equation (39), and update $\chi_{t+1}$ using Equation (40).32:    %***End precoding optimization***33: Check convergence.34:**end**

**Remark 3.**  *To enable joint optimization in ISAC systems, the conventional WMMSE formulation is extended to incorporate a sensing beamforming objective. The transmit precoding matrix accurately directs the signal energy toward the target users, thereby improving their SINRs and suppressing inter-user interference. For sensing, the transmit beam is spatially shaped to concentrate energy on the intended targets or regions, enhancing target detection accuracy. The additional sensing term results in a modified eigenstructure compared to the classical WMMSE framework, which steers the transmit energy towards target directions while preserving communication performance.*

### 3.3. From Sensing Beam Gain to Angle-Oriented Sensing

Based on the original sensing beam gain optimization framework, the proposed design’s sensing capability is further expanded and analyzed at the task level. Specifically, improving the sensing beam gain in the target direction directly enhances the received echo power and angular resolution. The beam alignment process naturally generates Angle-of-Arrival (AoA) and Angle-of-Departure (AoD) measurements, and the communication beam management directly supports the sensing functionality [37]. Consequently, we further evaluate the system performance in the target detection and angle estimation tasks.

While the sensing objective in Equation (19) is explicitly formulated to maximize the sensing beam gain, this design inherently facilitates more sophisticated angle-domain sensing functionalities. Based on the radar signal model presented in Equations (15)–(18), the received echo power along any specific direction is strictly governed by its corresponding sensing beam gain. Consequently, boosting the sensing beam gain not only enhances the target detection capabilities, but also lays a solid foundation for advanced angle-dependent tasks.

From Equation (16), the output sensing signal-to-noise ratio of the target unit is given by:

(41) $$\gamma_{s}\left(\phi_{r}\right)=\frac{{\left|\beta_{r}\right|}^{2}h_{\mathrm{rad}}^{H}\left(\phi_{r},\phi_{p_{L}}\right)R_{s}h_{\mathrm{rad}}\left(\phi_{r},\phi_{p_{L}}\right)}{\sigma_{n}^{2}}$$

To further characterize this property, the transmit covariance-induced angular sensing spectrum is defined as:

(42) $$S_{\mathrm{ang}}\left(\phi_{r}\right)=h_{\mathrm{rad}}^{H}\left(\phi_{r},\phi_{p_{L}}\right)R_{s}h_{\mathrm{rad}}\left(\phi_{r},\phi_{p_{L}}\right)$$

From this perspective, the proposed joint optimization scheme can be interpreted as enhancing the energy concentration within the target angular region while maintaining communication performance. The optimized sensing beam pattern provides stronger support for subsequent angle-domain sensing tasks, including detection probability, angular discrimination, and angle estimation.

Based on the above observation, an auxiliary angle-oriented sensing metric is formulated as:

(43) $$J_{\mathrm{ang}}=\int_{\Theta_{r}}S_{\mathrm{ang}}\left(\phi_{r}\right)d\vartheta -\varsigma \int_{\Theta_{r}^{c}}S_{\mathrm{ang}}\left(\phi_{r}\right)d\vartheta$$

where $\Theta_{r}$ represents the target angle region, $\Theta_{r}^{c}$ denotes the angular region outside the desired sector, and ς is a weighting coefficient used to compensate for the angular leakage outside the desired sector. This statement provides an angle-oriented explanation for the sensing beam gain.

At angle $\phi_{r}$, the complex output obtained through the angular domain scanning is $t\left(\phi_{r}\right)$. Under the null hypothesis $H_{0}$, the detection unit only contains noise. On the other hand, under the alternative hypothesis $H_{1}$, the detection unit simultaneously contains the target signal and noise. For any given angular unit to be tested, consider the following binary hypothesis testing problem:

(44) $$\begin{array}{l} H_{0}:t\left(\phi_{r}\right)=n\left(\phi_{r}\right),\mathrm{target}\;\mathrm{absent}\\ H_{1}:t\left(\phi_{r}\right)={\hat{h}}_{\mathrm{rad}}\left(\phi_{r}\right)+n\left(\phi_{r}\right),\mathrm{target}\;\mathrm{present} \end{array}$$

where under the null hypothesis, the noise $n\left(\phi_{r}\right)∼CN\left(0,\sigma^{2}\right)$ is a zero-mean complex Gaussian variable, and similarly, $t\left(\phi_{r}\right)$ is a zero-mean complex Gaussian variable, ${\hat{h}}_{\mathrm{rad}}\left(\phi_{r}\right)$ is a scalar gain. This work uses the square of the observed quantity $T\left(\phi_{r}\right)={\left|t\left(\phi_{r}\right)\right|}^{2}$ as the decision statistic. The false alarm probability $P_{\mathrm{FA}}$ can be obtained:

(45) $$P_{\mathrm{FA}}=\mathrm{Pr}\left(T\left(\phi_{r}\right)>\eta |H_{0}\right)=\exp\left(\frac{-\eta }{\sigma^{2}}\right)$$

Under the alternative hypothesis $H_{1}$, the target echo $t\left(\phi_{r}\right)$ is regarded as a deterministic complex amplitude. The decision statistic follows a non-central chi-square distribution under $H_{1}$. The detection probability is expressed in the form of a first-order Marcum Q function as:

(46) $$P_{D}=Q_{1}\left(\sqrt{\frac{2{\left|\beta_{r}\right|}^{2}S_{\mathrm{ang}}\left(\phi_{r}\right)}{\sigma^{2}}},\sqrt{\frac{2\eta }{\sigma^{2}}}\right)$$

Note that when the threshold and noise power are fixed, the detection probability $P_{D}$ is directly determined by the sensing beam gain in the target direction.

The AOA of the target is estimated from the detected spectral peak, which is obtained using the peak search method. Then, the angle estimation value of the *r*th target:

(47) $${\hat{\phi }}_{r}=\arg\underset{\phi \in \Theta }{\max}\;S_{\mathrm{ang}}\left(\phi_{r}\right)$$

**Remark 4.**  *In the framework of binary hypothesis testing, increasing sensing energy toward the target direction directly increases the probability that the decision statistic exceeds the detection threshold, thereby improving target detection probability. Therefore, the increase in the sensing beam gain can be rigorously interpreted as a sufficient statistical basis for improvement in detection probability.*

### 3.4. Analysis on Computational Complexity

The optimization scheme for the hybrid RIS-assisted ISAC system employs an alternating framework to jointly refine the complex reflection coefficient matrix $\Phi_{t}$ and BS transmit precoding matrix F. It maximizes a weighted sum of communication rate R and sensing beam gain P, modulated by a communication-sensing controllable coefficient α, subject to a total BS transmit power constraint. The framework utilizes $T_{\Phi }$ and *T***_F_** iterations individually until convergence, with system parameters including *M* BS-antennas, *K* single-antenna-users, *N* sensing-targets, and *L* RIS elements.

(1) Manifold step for updating Φ

In the subroutine for optimizing RIS phase shift matrix Θ with fixed F, the Riemannian Hessian Algorithm is used on complex unit circle manifold. The primary computations include evaluating the objective function and its gradients: forming the equivalent channel incurs $O\left(LKM+NLM\right)$ operations, computing SINR and communication rate R adds $O\left(K^{2}M\right)$, the sensing gain P requires $O\left(NLM+NKM\right)$; deriving the Euclidean gradient $\nabla_{\theta }f$ dominates with $O\left(LM\left(K+N\right)\right)$. Incorporating the second-order information, the Riemannian Hessian computation escalates to $O\left(L^{3}\right)$ if a full Hessian operation is formed, though approximations like quasi-Newton method can reduce this to $O\left(L^{2}\right)$ per iteration. Update and adaptive step-size adjustment contribute negligible $O\left(L\right)$. Thus, the almost computational complexity leads to a total of $O\left(T_{\Phi }LM\left(K+N\right)\right)$ for this subroutine.

(2) Lagrangian steps for updating F

For optimizing the precoding matrix F with fixed Φ, the Lagrange multiplier method is employed, augmented by Newton’s method for solving dual variables. Key steps involve evaluating mean-squared errors and optimal weights at $O\left(KM^{2}\right)$, and solving the partial derivatives via eigenvalue decomposition of matrix, costing $O\left(MK^{2}+K^{3}\right)$ per user or collectively $O\left(KM^{3}\right)$. The power expression and Newton’s update add $O\left(MK\right)$ terms. Thus, the additional computational complexity leads to a total of $O\left(T_{F}\left(KM^{2}+M^{3}\right)\right)$ for this subroutine.

(3) Expand the task of sensing spatial orientation

The additional computational complexity brought about by task-level sensing extension mainly comes from three steps: spectral evaluation, detection based on CA-CFAR, and local AOA estimation. Firstly, the system needs to calculate point by point on the angle grid. For a single point, the computational complexity is $O\left(M^{2}+ML\right)$. This angular area is divided into G discrete grids. Then, the complexity of constructing the angular domain spectrum is $O\left(G\left(M^{2}+ML\right)\right)$. Subsequently, in the corner detection stage, the CA-CFAR scheme is employed and executed on the spectrum with a complexity of $O\left(GN_{\mathrm{tr}}\right)$. Among them, the definition of G and $N_{\mathrm{tr}}$ is presented in. The final area and peak search AOA estimation complexity is $O\left(1\right)$. Therefore, the complexity of expanding spatial orientation sensing is mainly determined by $O\left(GM\left(M+L\right)\right)$.

## 4. Simulation Experiments and Analysis

This section validates the proposed scheme and compares it with the popular methods: dual-function radar communication system without RIS, i.e., an architecture that relies solely on the BS side for communication and sensing [40]; passive-only RIS system where only the phases are controllable without amplification and a BS beam [41]; random phase RIS system where the required phases are randomly arranged [42]; active-only RIS system that employs an active RIS [43].

To rigorously evaluate the average performance and illustrate the statistical variance, multiple independent Monte Carlo trials were conducted. For a fair comparison, all considered schemes employ an identical number of reflecting elements. The fundamental difference between the active and passive elements is captured by the amplitude constraints imposed on their respective reflection coefficients. This article uses MATLAB (version 2025) to conduct the simulation. The important parameter settings are shown in Table 1.

In the simulations, we assume a three-dimensional Cartesian coordinate system where the BS and the RIS are deployed at (0,0,25) m and (15,0,25) m, respectively. The users are uniformly distributed within the area of [70, 90] m × [−10, 10] m at a height of 1.5 m. Furthermore, we adopt the close-in (CI) free-space reference model to characterize the large-scale path loss. The shadowing standard deviations are configured as 2 dB for the BS-RIS link, 4 dB for the RIS-user links, and 8 dB for the direct BS-user links.

**Figure 3:**
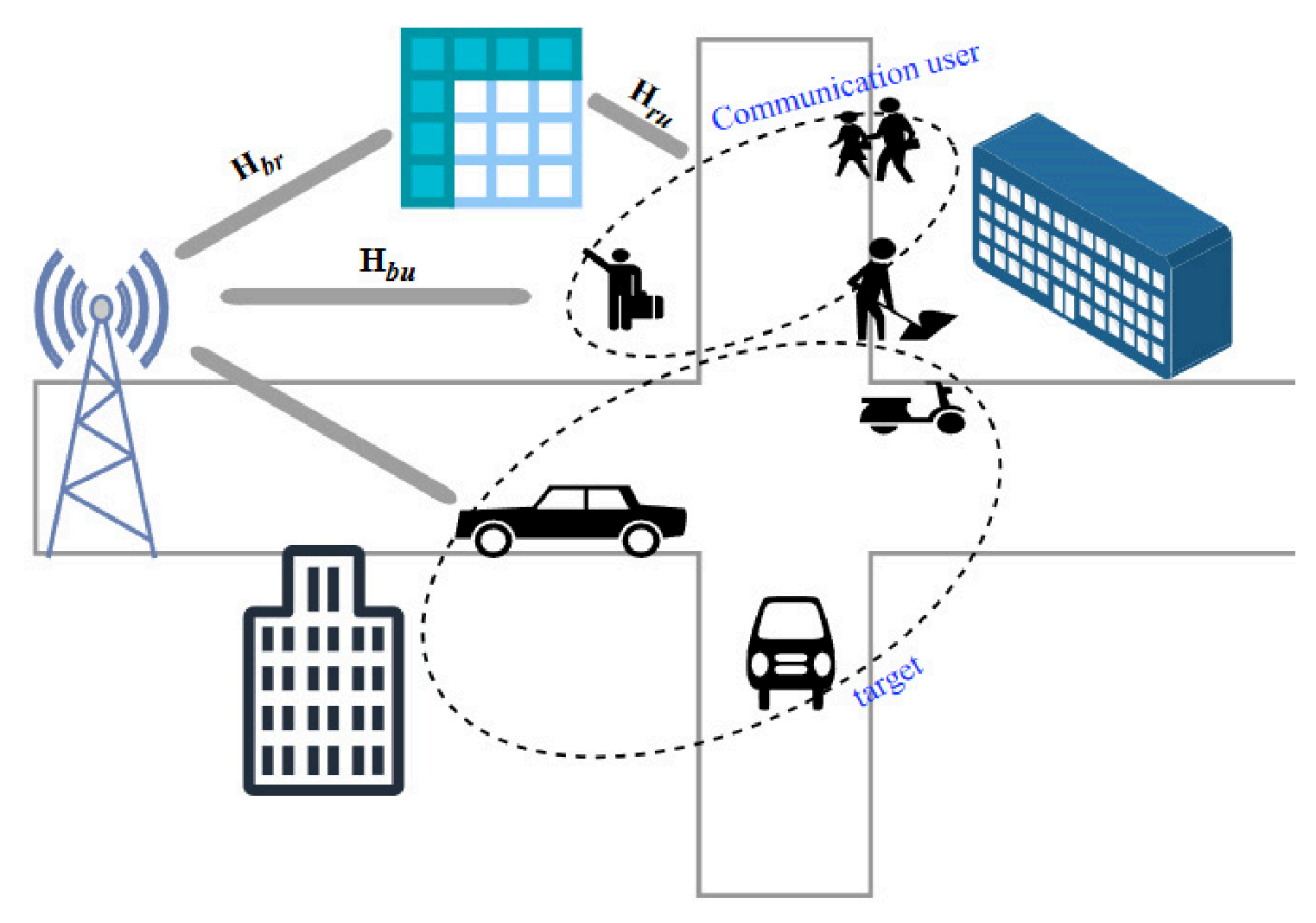
Simulation Scenario Description.

To further evaluate the angle estimation performance at the task level, this paper constructs multi-target angle sensing scenarios within the predefined angular domain, as shown in Figure 3. The angular scanning range is set to [−60°, 60°], and the three sensing targets are located at −25°, 5°, and 30. Each target is characterized as a scattering cluster consisting of multiple scattering points, whose principal axis is defined as the ground-truth AoA. By jointly optimizing the communication and sensing objectives, the extended WMMSE algorithm yields a transmit precoding matrix that optimally balances the spatial directions of both the user channels and the targets. As a result, the spatial degrees of freedom can be leveraged to focus signal energy on the sensing targets without compromising communication requirements.

**Figure 4:**
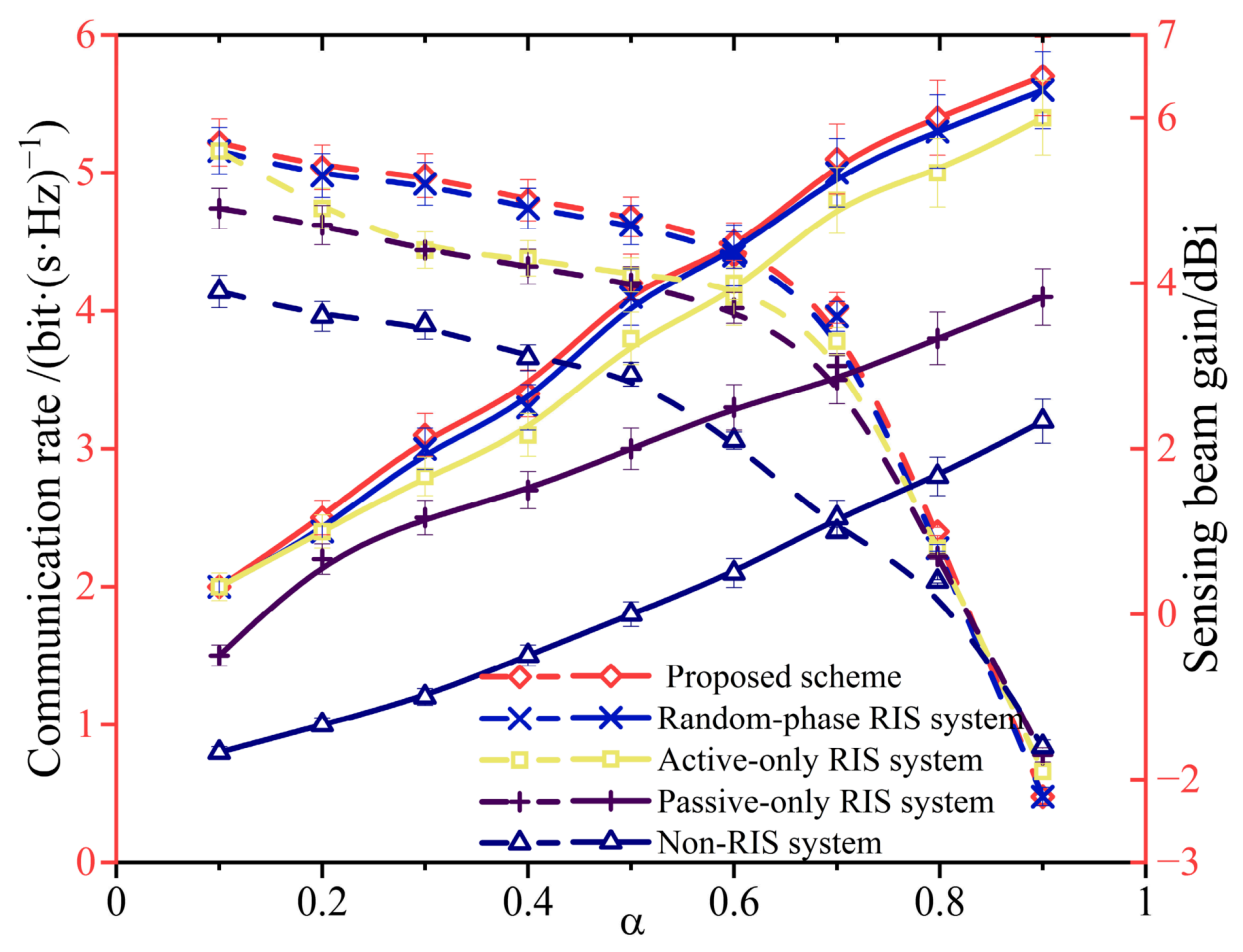
Communication-Sensing Trade-off across Schemes.

Figure 4 shows the communication-sensing trade-off curves for five schemes at an SNR of 9 dB, where the solid lines denote communication rate and the dashed lines represent sensing gain. Notably, the proposed scheme and the random-phase RIS deliver the best joint performance over most operating points. In the high-rate region of *α*, the proposed design degrades more gracefully. At a communication rate of 5 bit·(s·Hz)^−1^, the proposed scheme still achieves a sensing beam gain of 4.8 dBi, whereas the baselines drop markedly. Although the random-phase RIS attains slightly higher gain at a few isolated points, the proposed scheme yields a smoother, more stable frontier and demonstrates superior capability when simultaneously pursuing high communication rate and sensing gain.

**Figure 5:**
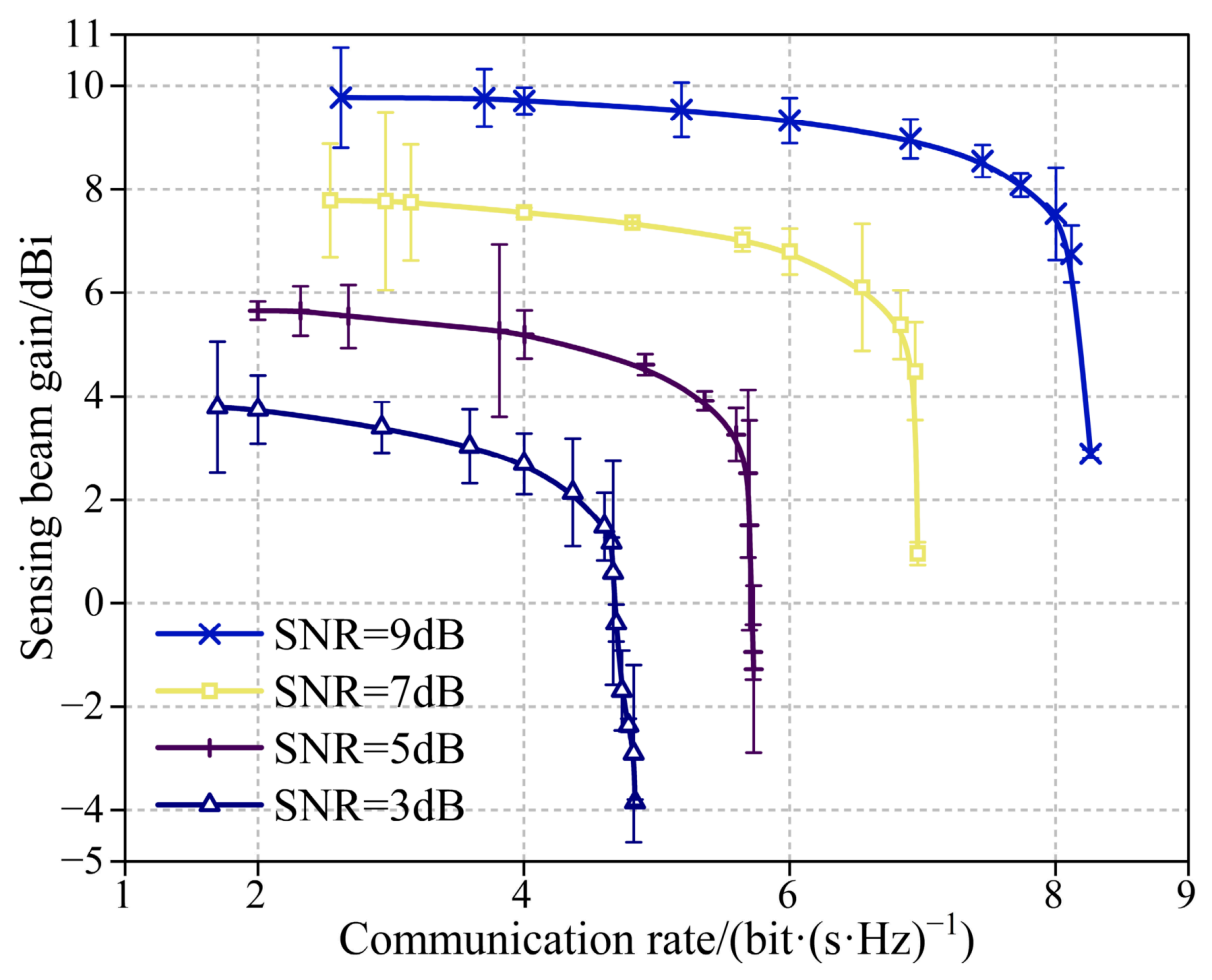
Communication-Sensing Trade-off under SNR.

Figure 5 shows the communication–sensing trade-off curves under different transmit SNRs. As the SNR increases from 3 dB to 9 dB, the curves shift upward and to the right, yielding higher sensing beam gain at a given communication rate and a higher rate under the same sensing requirement. At 9 dB, the curve remains nearly flat over the rate range of 7 to 8 bit·(s·Hz)^−1^, sustaining a sensing gain of approximately 7 to 9 dBi. When the SNR decreases to 7 dB, the knee point extends to around 6 bit·(s·Hz)^−1^ with a corresponding sensing gain of roughly 7 dBi. At 5 dB, the knee appears earlier, near 5.3 bit·(s·Hz)^−1^, followed by a steep decline toward 0 dBi. For the 3 dB case, the system is clearly noise-limited and the sensing gain drops below 0 dBi even at moderate rates. These results indicate that SNR governs whether the system operates in the noise-limited or power-limited regime. Further, the higher SNR enlarges the region where both communication rate and sensing gain can be achieved concurrently.

**Figure 6:**
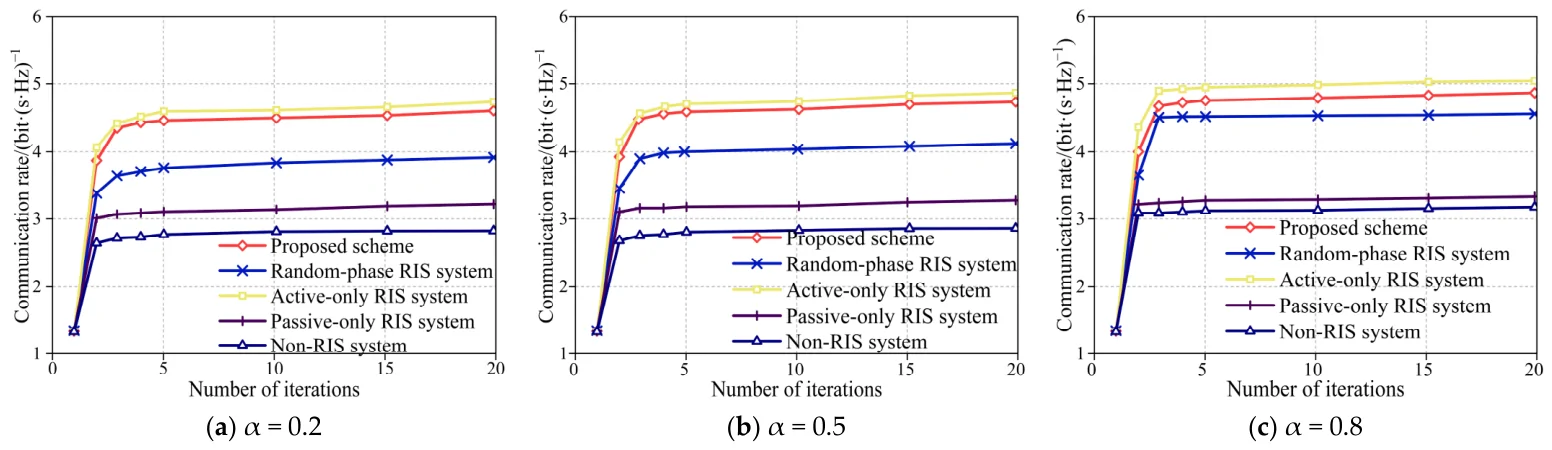
Convergence of Communication Rate under α.

Figure 6 shows the convergence of communication rate at an SNR of 9 dB under different values of the controllable coefficient α. All evaluated methods exhibit a monotonically increasing trend with from four to six iterations. The communication rate rises with α, which reflects the communication-oriented trade-off yields a higher plateau. The proposed scheme consistently achieves the highest plateau and smoother late-stage improvement. The active-only RIS system achieves the second-best performance, but its gain saturates due to noise amplification. Both the passive-only RIS and random-phase RIS systems converge to significantly lower rates, whereas the non-RIS baseline yields the worst performance. Equation (16) implies that the smaller α allocates more degrees of freedom to sensing beam gain, whereas the larger α concentrates power on the dominant singular mode, increasing capacity. By employing the Lagrange multiplier method and Newton’s method, the objective value is guaranteed to be non-decreasing at each iteration, hence the observed rapid monotonic convergence. The results indicate that the proposed design remains superior in the sensing-oriented, balance-oriented, and communication-oriented regimes.

**Figure 7:**
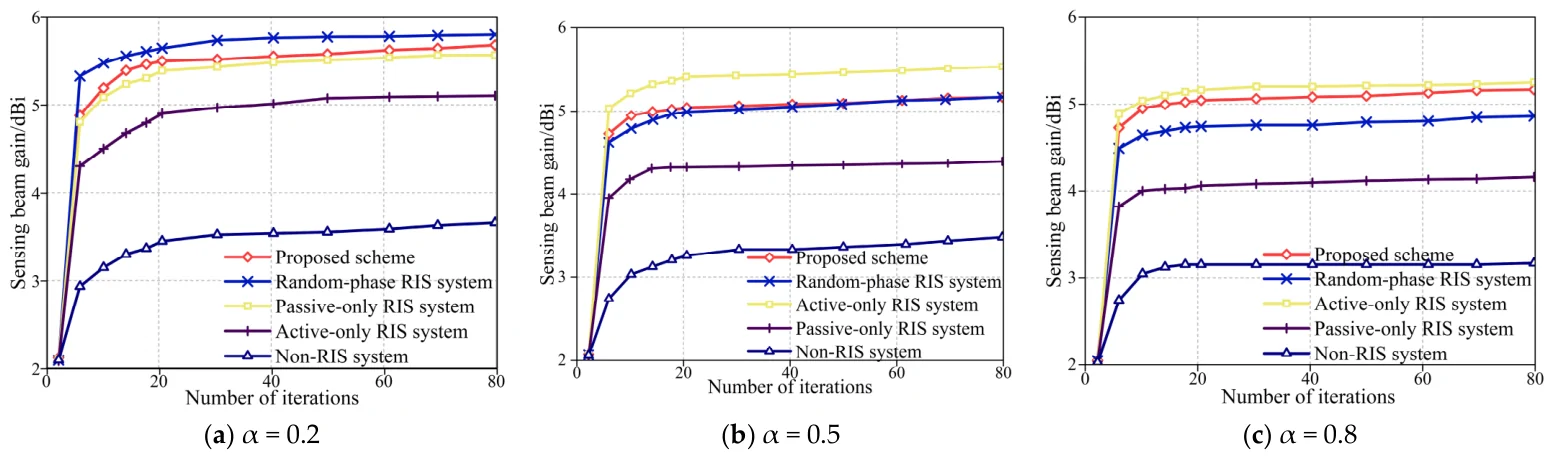
Convergence of Sensing Beam Gain under α.

Figure 7 shows the convergence of sensing beam gain at an SNR of 9 dB for different values of *α*. All considered schemes rise rapidly within the first five iterations, then plateau after about 20 iterations, consistent with the monotonic convergence of the alternating optimization approach. In the steady state, the active-only RIS system and passive-only RIS system achieve the highest gains with a small gap. The proposed scheme closely follows and maintains a gain level comparable to the top two baselines while accounting for the communication objective, which demonstrates the robust preservation of sensing performance. The random-phase RIS system and non-RIS system remain almost flat and substantially lower, indicating the lack of controllable degrees of freedom. As the weight *α* moves among three values, the plateau level shows only a moderate adjustment. Specifically, the proposed scheme sustains roughly 5 dBi when α is 0.2. There is a slight drop when *α* is 0.5 due to resource reallocation toward the communication rate. Ultimately, the high sensing beam gain achieved by the proposed scheme corroborates its superior joint performance with the communication rate.

**Figure 8:**
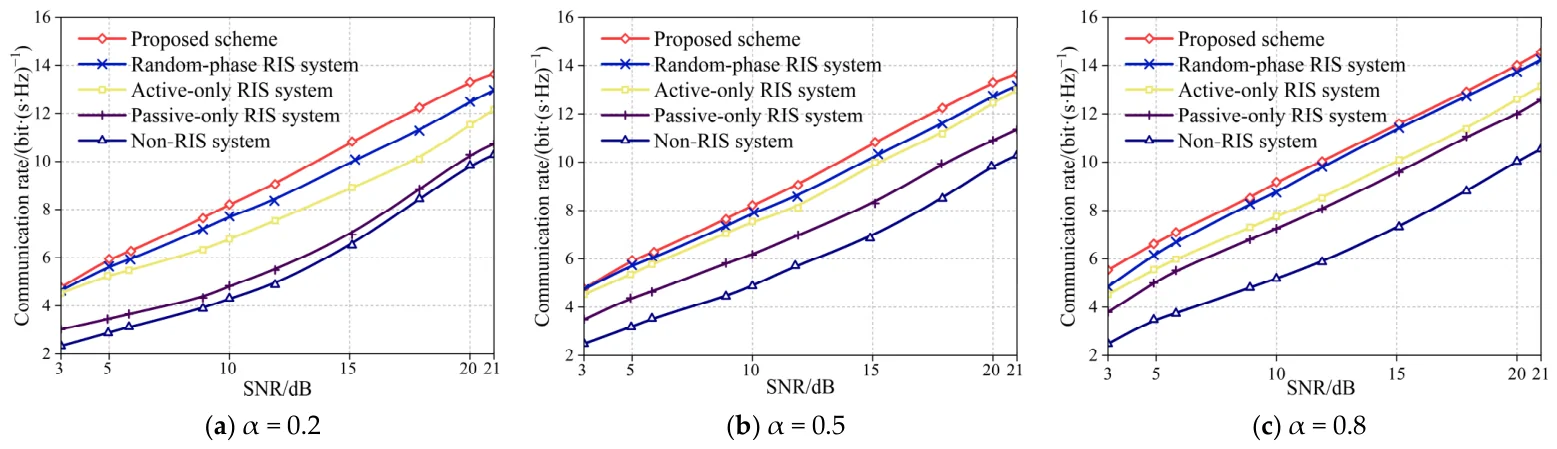
Communication Rate across SNR under α.

Figure 8 shows the communication rate versus the SNR for different values of α. The rate scales nearly linearly with SNR. The proposed scheme consistently achieves the highest performance tier and robustly outperforms the active-only, passive-only, and non-RIS baselines. Compared with the random-phase RIS system, it exhibits only a marginal rate gap while satisfying the sensing requirement. Increasing α from 0.2 to 0.8 shifts all curves upward without changing the relative ordering. Moreover, it evidences the scalability and robustness of the joint optimization. The active-only RIS system is constrained by noise amplification at low-to-middle SNRs and fails to catch up at high SNRs, whereas the passive-only RIS system and non-RIS system are limited by their aperture and degrees of freedom. Overall, the proposed scheme achieves a near Pareto-optimal balance over wide SNR and weighting regimes.

**Figure 9:**
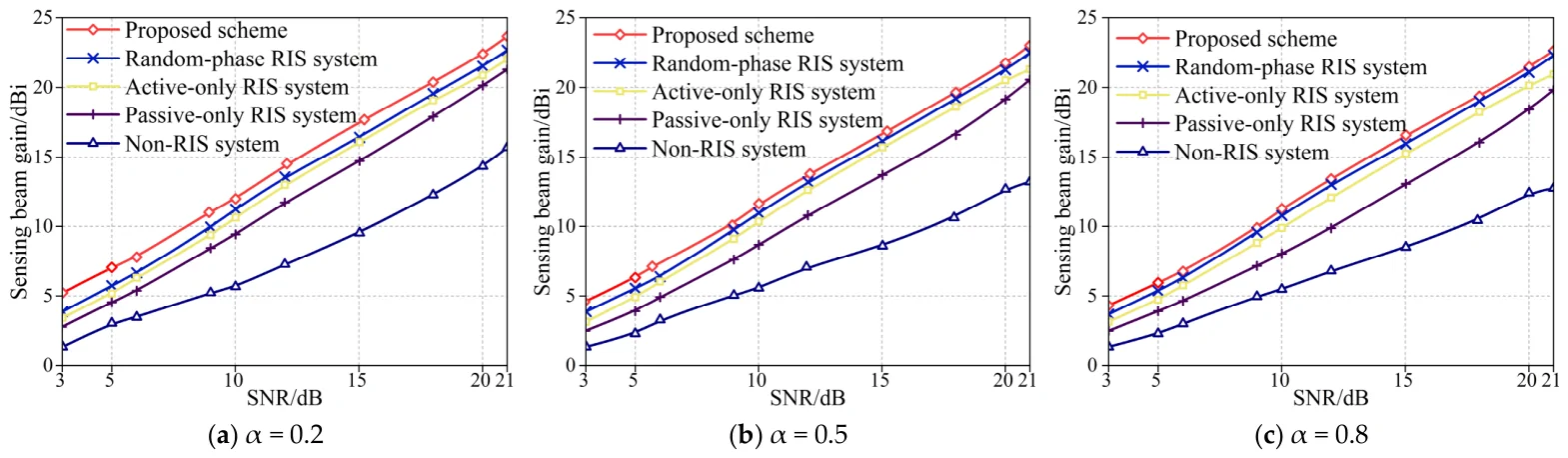
Sensing Beam Gain across SNR under α.

Figure 9 shows sensing beam gain versus the SNR for different values of *α*. The gain increases monotonically and almost linearly with the SNR. The relative ordering of the schemes remains invariant. The proposed scheme consistently maintains the highest gain, followed by the random-phase RIS system and the active-only RIS system. The passive-only RIS system and the non-RIS system exhibit the lowest gains. As *α* increases from 0.2 to 0.8, the system shifts from sensing-oriented to communication-oriented. The curves show a slight downward shift at a given SNR. This is consistent with the weighted objective that prioritizes communication rate. Consequently, the reallocation of spatial resources reduces the degrees of freedom for beamforming and slightly decreases the sensing gain. The proposed optimization maintains a robust lead across all SNRs. This indicates that even under a strong rate bias, the proposed scheme can effectively preserve the main lobe while suppressing the sidelobes. The active-only RIS system suffers from noise amplification and hardware imperfections, whereas the passive-only RIS system lacks controllable gain. The random-phase RIS system, though sometimes close to the upper bound, does not guarantee compliance with the joint design objectives. Overall, the results show near-linear scalability with SNR and a stable performance advantage.

**Figure 10:**
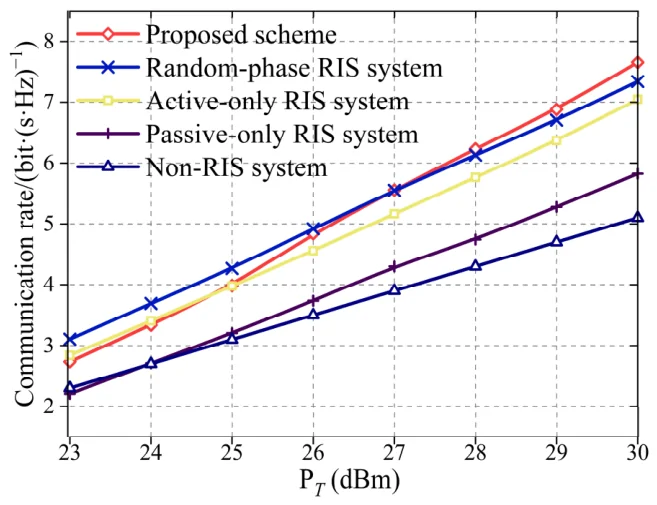
Communication rate variations of multiple schemes under the same power level.

Figure 10 shows the variation of communication rates for four RIS architectures with respect to the total power budget under the unified total power constraint. This experiment was conducted under the condition of an SNR of 9 dB, and the controllability coefficient α is 0.5. In the severely power-limited regime of 23 to 25 dBm, the random RIS baseline achieves the optimal rate. This is because its active elements are randomly distributed, resulting in lower dynamic power consumption, which in turn leaves a higher available transmit power for the BS. Conversely, in the relatively power-abundant regime of 28 to 30 dBm, the proposed scheme achieves the highest rate. The importance metric accurately locates the optimal positions for the active elements that achieve the maximum amplification gain. Furthermore, the hybrid RIS activates only a small number of active elements, which inherently results in lower power consumption compared to the fully active RIS. Under a unified power budget, the hybrid RIS still outperforms the other baselines in terms of the achievable communication rate.

**Figure 11:**
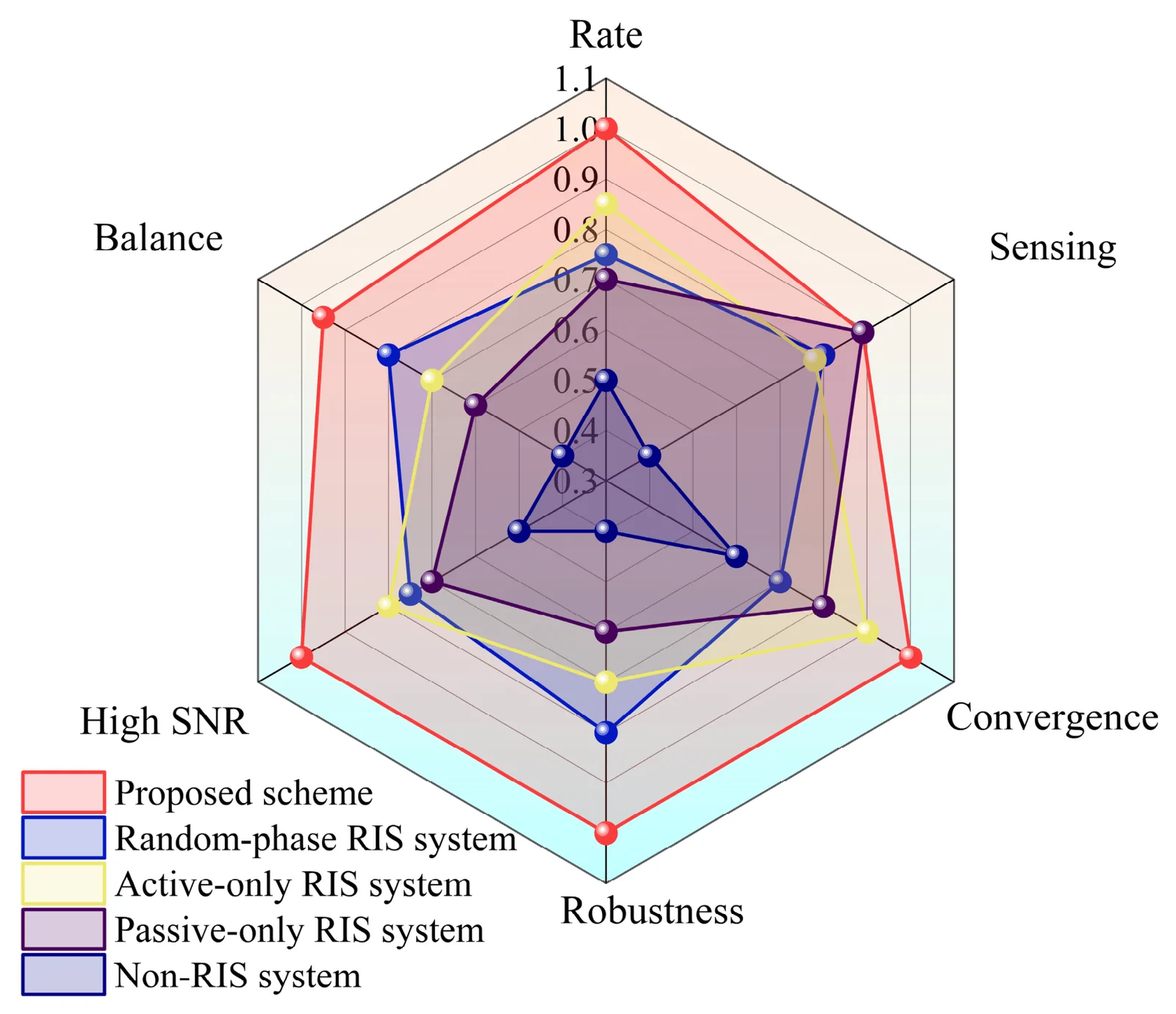
Radar Chart across Schemes.

Figure 11 shows the radar chart that provides a comparative analysis of all-round performance of different configurations in a multi-user and multi-target environment under the number of active reflecting elements is 8, SNR is 9 dB, and *α* is 0.5. Among them, there are six quantitatively defined indicators, each of which is normalized to [0, 1] for fair comparison. Some definitions are as in Table 2.

Among them, define $\sigma_{R}$ as the standard deviation of the rate, and s as the growth rate of the rate R when linearly fitted with SNR under high signal-to-noise ratio conditions. The axes represent key performance metrics, including rate, balance, high SNR, robustness, sensing, and convergence. The proposed scheme outperforms the other methods in nearly all aspects, particularly in rate, sensing, and robustness. It achieves a well-balanced performance, achieving high values in both the communication rate and the sensing gain, which are critical in an ISAC system. Additionally, the proposed system demonstrates excellent convergence, indicating the stability and efficiency of the optimization process.

**Figure 12:**
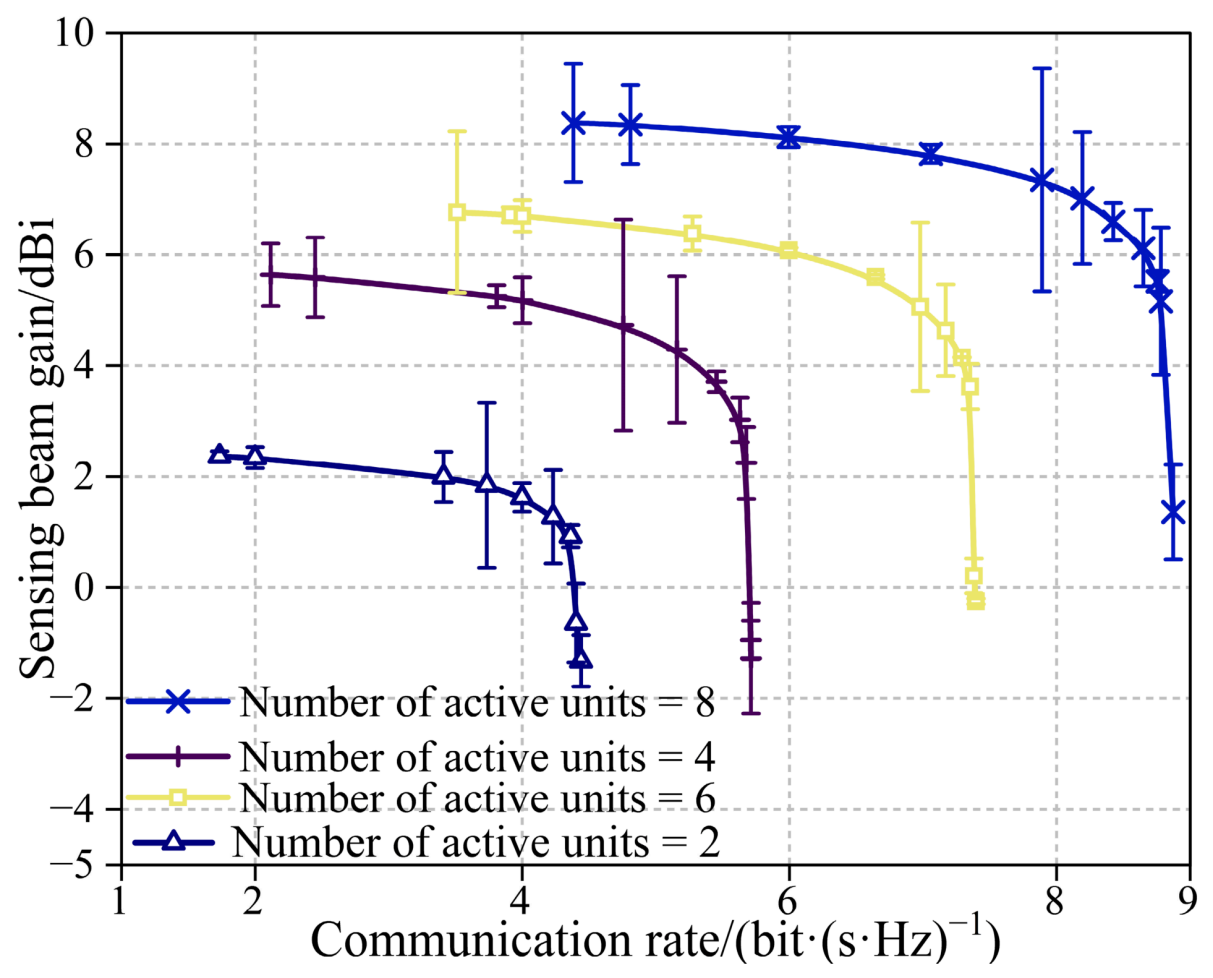
Communication-Sensing Trade-off across Numbers of Active Reflecting elements.

Figure 12 shows the number of active reflecting elements on the communication-sensing trade-off curves at an SNR of 9 dB. As the number of RIS elements increases from 2 to 8, both the communication rate and the sensing gain values increase. This indicates higher sensing beam gain at a given rate or a higher communication rate under the same sensing requirement. When the number of active elements is 8, and the communication rate is 7 to 8 bit·(s·Hz)^−1^, the sensing gain can still remain at 5 to 8 dBi. When the number of elements is 2, the inflection point occurs around 3.8 bit·(s·Hz)^−1^, and then the sensing gain rapidly drops below 0 dBi. The outward shift of the trade-off curve when the number of active elements increases from 2 to 4 is the most pronounced, which reflects the combined benefits of aperture gain and additional beam degrees of freedom. The marginal improvement when the number of active elements increases from 6 to 8 is smaller. The results indicate that the RIS aperture can simultaneously enhance communication capacity and sensing resolution under both AWGN and multipath-limited conditions.

**Figure 13:**
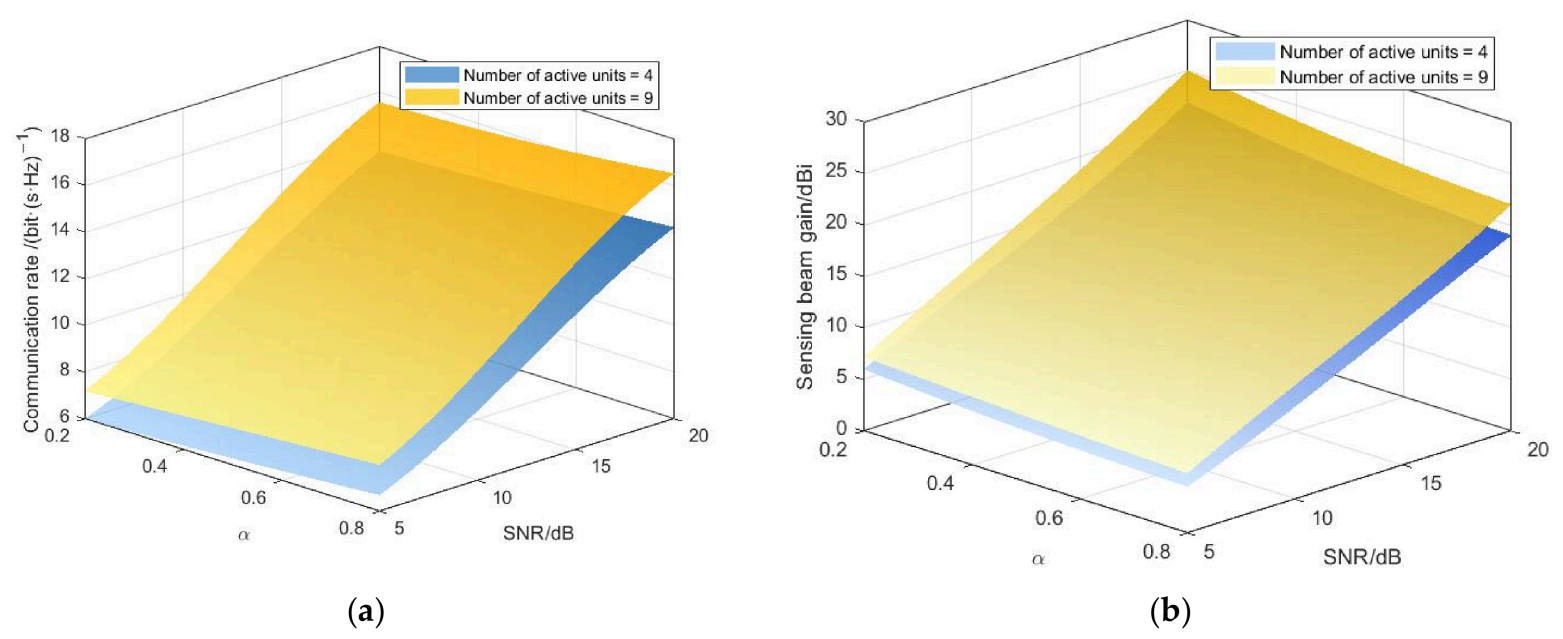
**3**D Surfaces of (**a**) Communication Rate and (**b**) Sensing Beam Gain versus α and SNR.

Figure 13 shows the joint variation of the communication rate and the sensing beam gain with respect to *α* and the SNR. Note that both metrics increase monotonically with SNR. However, the sensing beam gain exhibits a higher sensitivity to α, shifting downward as α increases to prioritize the communication rate. In contrast, the communication rate shows only a moderate dependence on α sustaining its improvement across the entire domain. Furthermore, increasing the number of active reflecting elements (9 elements versus 4 elements) uniformly elevates both metrics over the entire (*α* and SNR) plane. This advantage becomes more pronounced at medium-to-high SNRs, reflecting the enhanced array gain provided by a larger reflecting aperture. The proposed scheme offers desirable tunability and robustness. It can be explained that *α* enables a continuous and controllable communication-sensing trade-off and scaling the RIS raises overall performance.

Table 3 shows the comparison of the costs and performance under specific conditions for the four baseline schemes mentioned in the paper. The unit for communication performance is bit·(s·Hz)^−1^, and the unit for sensing performance is dBi. For the Active-only RIS, there is no unit modulus constraint allowing for simpler Euclidean updates, which can avoid the need for manifold projection and second-order Riemannian operations. For the Passive-only RIS, the hybrid scheme must operate on the Riemannian manifold and simultaneously handle the amplitude constraints and activation masks of active elements.

To transition from sensing-beam gain to angle-sensing experiments, we adopt the following procedure. First, we conduct an angular scanning simulation to verify whether the proposed beam design can successfully illuminate and localize the target directions. Based on the scanning outputs, we employ the CA-CFAR detector with a predefined false-alarm probability to quantitatively assess target detection performance. Finally, conditioned on successful detection, the accuracy of AoA estimation is evaluated in terms of the root-mean-square error (RMSE). In this way, the sensing performance is assessed from three complementary perspectives: qualitative target visualization, statistical detection probability, and quantitative estimation error. To verify the system’s ability to support communication services concurrently with sensing, we introduce a symbol transmission simulation. While the BS performs the angle-oriented sensing task, it simultaneously transmits 16-QAM symbols to the communication users. The communication link quality is then evaluated via constellation diagrams [44].

**Figure 14:**
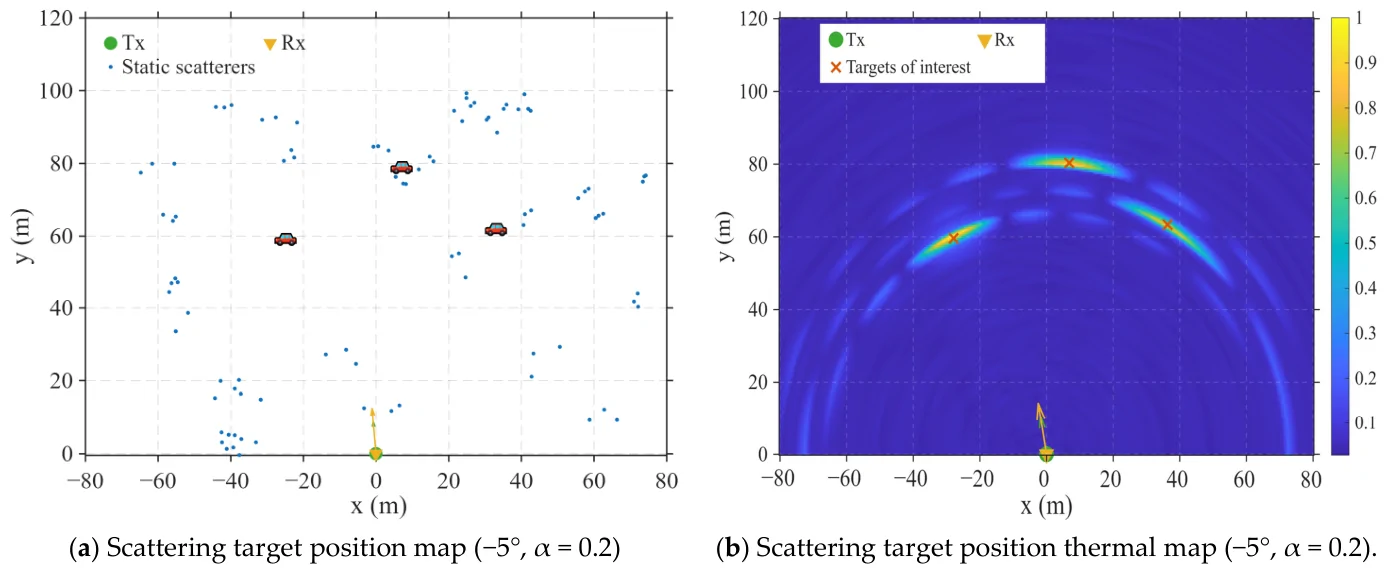
Scattering target detection.

Figure 14 shows the results of the angle-oriented sensing simulation. Figure 14a shows the targets and randomly dispersed scattering points in a two-dimensional coordinate system. The BS is pointing at −5°, and the three sensing targets are located at −25°, 5°, and 30°. Figure 14b shows the 2D spatial heatmap of the sensing response. The brighter region indicates a higher response value. Thus, the stronger received echo power at that angle. In this simulation, the controllable coefficient α is set to 0.2. The system is mainly focused on sensing tasks. As a result, all three pre-defined targets are successfully illuminated and detected.

**Figure 15:**
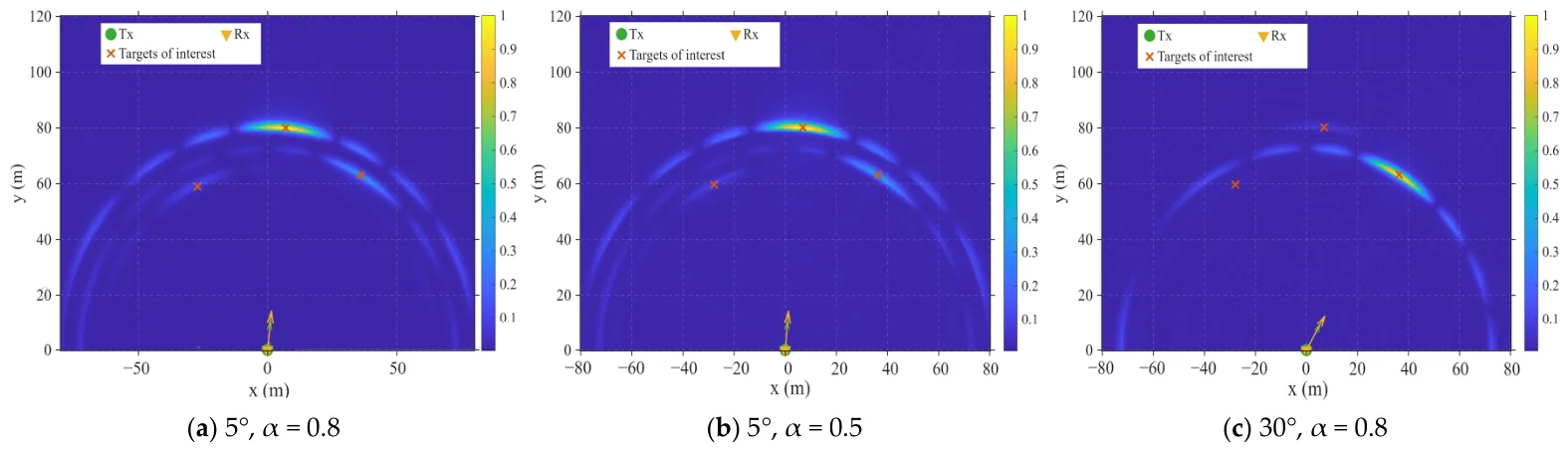
Scattering target detection; scattering target position thermal map.

Figure 15 shows the spatial heatmaps of the scattering targets under different sensing directions and different controllable coefficient α. The high-intensity regions in the heatmaps accurately align with the pre-designed target directions. This indicates that the joint optimization strategy effectively focuses transmit energy toward the target angular domain, enhancing the sensing echo response while guaranteeing communication performance. From the comparison of Figure 15a,b for the same target direction of 5°, the high-energy regions become significantly more concentrated when controllable coefficient α decreases from 0.8 to 0.5. This confirms that allocating more spatial degrees of freedom to sensing directly enhances the target detectability. Figure 15c demonstrates that when the main direction of the target is adjusted to 30°, the system can still accurately scan the target within the corresponding angular domain.

**Figure 16:**
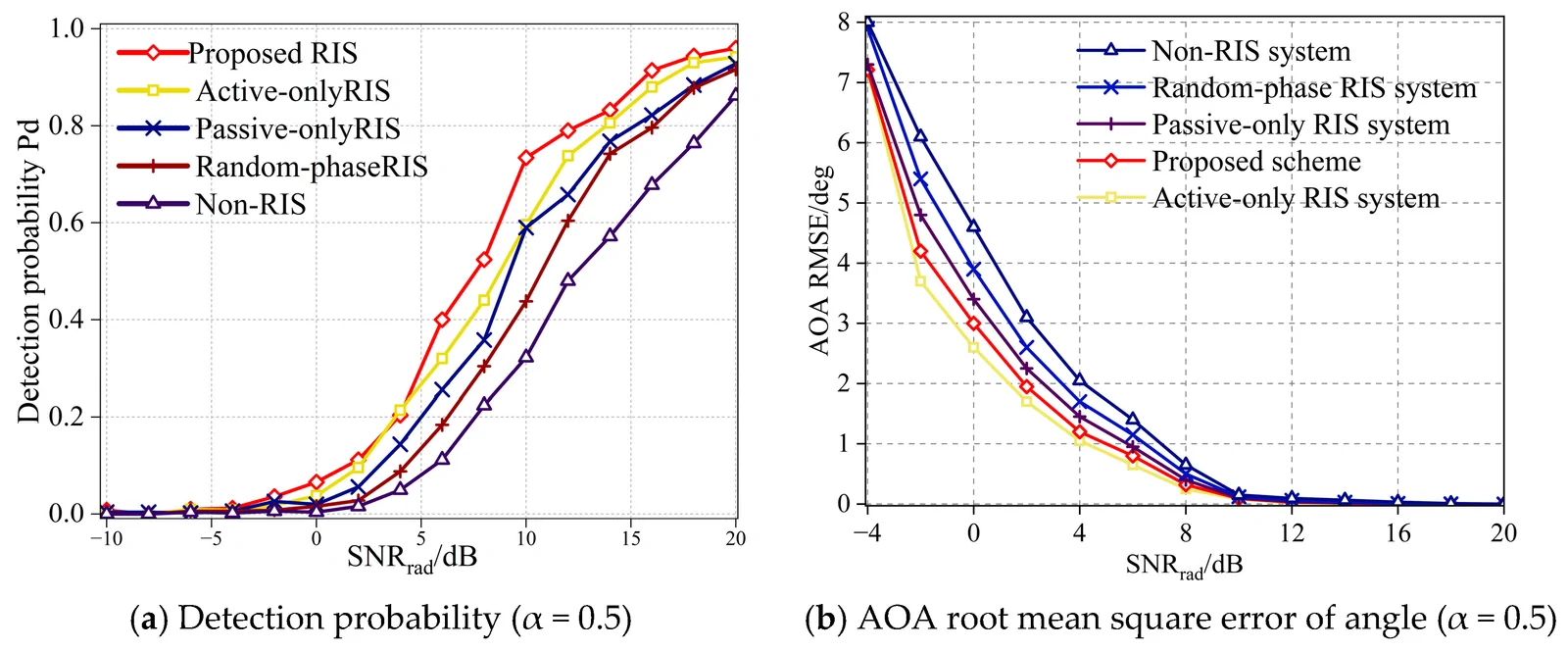
Detection result.

Figure 16 shows the target detection probability and the RMSE of the AoA estimation versus the sensing SNR. Specifically, Figure 16a shows the detection probability for different RIS structures. The controllable coefficient α is set to 0.5. As the sensing SNR increases, the detection probability of each scheme gradually rises. All curves eventually converge to a similar level. Among them, a higher signal-to-noise ratio enhances the distinguishability between the signal existence hypothesis $H_{1}$ and the noise hypothesis $H_{0}$. This increases the likelihood that the detection statistic exceeds the predetermined threshold. The hybrid RIS structure combining active-passive beamforming effectively enhances the gain of the beam towards the target direction. The competitiveness of the active RIS system is due to the fact that its active components provide additional signal amplification capability. Therefore, it provides more energy support for the sensing functionality. In the Figure 16b, note that as the sensing SNR increases, the AOA estimation error decreases rapidly. In the range from −4 dB to 2 dB, the signal power and noise can be distinguished, and the RMSE drops sharply. The proposed RIS scheme achieves a significantly lower AOA estimation error overall. This advantage stems from the joint optimization of the RIS phase shifts and the transmit precoding matrix. This joint optimization enhances the energy concentration within the target angular region.

**Figure 17:**
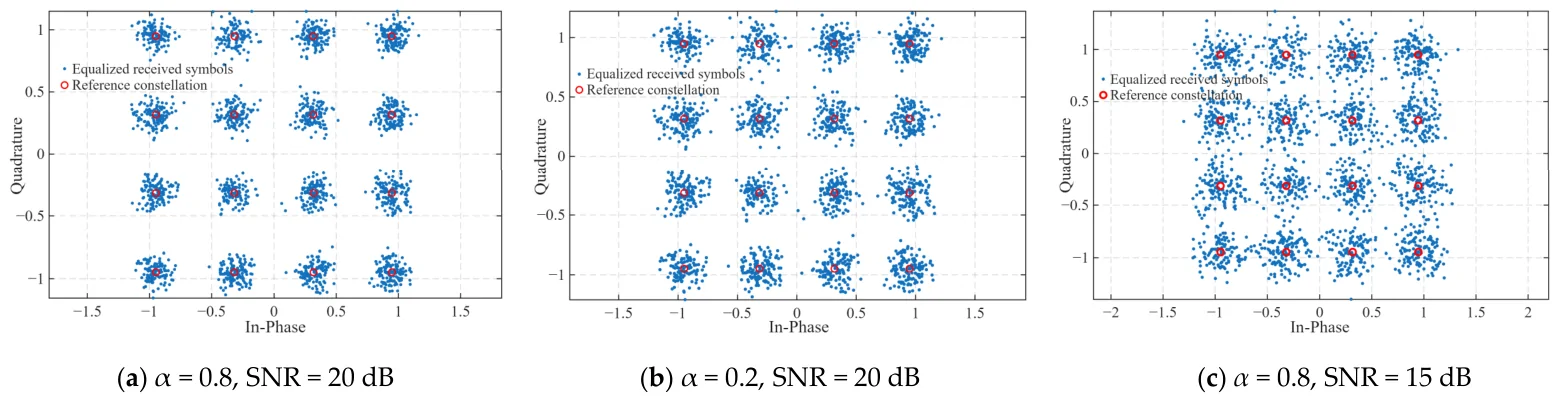
QAM constellation diagram.

Figure 17 shows the received 16-QAM constellation diagrams at two different SNRs. Note that the received symbols form clear clusters around the ideal constellation points. It indicates that the proposed joint optimization method can ensure the sensing capability while providing stable transmission performance for the communication link. In Figure 17a, given a controllable coefficient α of 0.8 and an SNR of 20 dB, the received symbols are closely concentrated near the reference [16]-QAM point. This result indicates that the system can effectively allocate spatial resources to maintain high-quality symbol recovery when communication plays a dominant role. It ensures the achievement of high communication fidelity in the integrated communication-sensing process. In Figure 17b, the controllable coefficient α is reduced to 0.2, and slight symbol dispersion appears in the constellation diagram. Even under a lower SNR condition, the received symbols still maintain a stable constellation clustering structure.

## 5. Conclusions

This paper proposes a hybrid RIS model to enhance the ISAC architecture by incorporating both active and passive reflecting elements. The design utilizes auxiliary fractional matrices and sub-model reflection modeling. Under the total power constraints, joint beam optimization is achieved by alternatingly designing the RIS phase shift matrix and the BS transmit precoding matrix. To maximize the weighted sum of the communication rate and the sensing beam gain, the locally optimal solutions are obtained to exhibit tunable multi-mode sensing capabilities. The key contribution is a novel RIS structure that balances active gain and passive phase, establishing the end-to-end MIMO-ISAC system model. When the BS precoding is fixed, the RIS phase shift matrix is adaptively updated on a complex circular manifold under unit-modulus constraints using a Riemannian Hessian algorithm. Conversely, with the RIS phase fixed, the BS precoding is optimized via the Lagrange multiplier method. For future work, we will further reduce computational complexity through algorithm acceleration and consider extensions to cooperative multi-point transmission and additional practical constraints.

## Figures and Tables

**Table 1:** Parameters on the Hybrid System.

Parameter	Value/Setting
Number of BS antennas *M*	8
Number of users *K*	4
Number of targets *N*	2, 3
RIS elements *L*	8*8
Number of active reflecting elements	8
Active elements gain factor *γ*	2
System bandwidth	10 MHz
Noise power *σ*^2^	–90 dBm
Maximum transmit power	30 dBm
Transmit SNR	3 dB, 5 dB, 7 dB, 9 dB
Weighting factor *α*	0.2, 0.5, 0.8
Channel model	Rayleigh fading with shadowing
Maximum number of iterations	80
Hyperparameter initial value	1
Hyperparameter threshold	10^−8^
Target scattering coefficient *β_r_*	0.5
Side leakage coefficient	0.1
Training Unit *N*_tr_	12
Discrete angle grid number G	241

**Table 2:** Indicator Definition.

Parameter	Description	Setting
Rate	Normalized average sum-rate	RRmax
Sensing	Normalized average sensing beam gain	PPmax
Robustness	Normalized robustness	RσR⋅ρmax
High SNR	Normalized high signal-to-noise ratio slope	ssmax
Convergence	Normalized convergence speed	$\max\left(T_{\Phi }+T_{F}\right)\left(\bar{T_{\Phi }}+\bar{T_{F}}\right)$
Balance	Harmonic mean of the rate and the perceptual gain	2RPRmaxPmaxRRmax+PPmax

**Table 3:** Baseline comparison (setting controllable coefficient to 0.5 and SNR of 15 as the benchmark).

Scheme	Communication Rate/Bit·(s·Hz)^−1^	Sensing Beam Gain/dBi
Proposed	10.8302	16.8576
Active-only RIS	10.3389	16.1431
Passive-only RIS	9.9943	15.7187
Random-phase RIS	8.2948	13.7167
Non-RIS	6.8568	8.6162

## Data Availability

The raw data supporting the conclusions of this article will be made available by the authors on request.

## Glossary

L	total number of reflecting elements in hybrid RIS	$y_{r}^{\mathrm{sens}}$	BS receiving sensing signal
$\delta_{i,j}$	importance score of RIS element at position	$y^{\mathrm{com}}$	signal received by user
$h_{ru,i,j}$	channel coefficient from RIS to user	α	communication-sensing controllable coefficient
$h_{br,i,j}$	channel coefficient from base station to RIS	$P_{T}$	maximum transmission power
p	priority index vector	$\beta_{r}$	target scattering coefficient
$d_{p}$	selection vector reordered by importance	$b\left(\phi_{R,r}\right)$	directional vector of RIS
$\gamma_{l}$	gain amplitudes of each element of RIS	$I_{M}$	*M*-dimensional unit matrix
Φ	complex reflection coefficient matrix for RIS	n	environmental noise received by BS
$\phi_{p_{i}}$	complex reflection coefficient of RIS	$E_{r}$	response matrix in N detection directions
θ	phase shift vector of RIS	$\mathrm{Hess}\left(\Phi_{t}\;\right)$	Riemannian Hessian on complex circle manifold
s	transmission symbol vector of user	$\eta_{t}\;$	Riemannian adaptive step size
$s_{k}$	transmission scalar symbol	$\xi_{t}$	step length attenuation coefficient
$a\left(\phi_{B,r}\right)$	BS receiving array vector	$a_{t}\left(\phi_{B,r}\right)$	BS transmission array vector
$H_{bu}$	channel matrix from BS to user	$\zeta_{t}$	hyperparameter balancing between gradient and curvature
$H_{br}$	channel matrix from BS to RIS	$e_{k}$	mean squared error
$H_{ru}$	channel matrix from RIS to user	$\omega_{k}$	optimal weight
$H_{b}$	equivalent channel matrix	χ	Lagrange multiplier
w	additive white Gaussian noise	A	composite matrix balancing sensing and communication
x	transmission signal of user	$b_{k}$	effective goal-oriented vector for user *k*
F	transmission precoding matrix	$c_{k}$	projection vector of eigenspace of matrix
$f_{k}$	transmission precoding vector for user *k*	$\gamma_{s}\left(\phi_{r}\right)$	output sensing SNR
$u_{k}$	optimal receive equalizer for user *k*	$S_{\mathrm{ang}}\left(\phi_{r}\right)$	transmission covariance-induced angular sensing spectrum
$\sigma_{\mathrm{act}}$	thermal noise of active components	$J_{\mathrm{ang}}$	directional perception effectiveness index of angle
$\gamma_{k}$	SINR for user *k*	$\Theta_{r}$	target angle region
R	Achievable sum rate	$P_{\mathrm{FA}}$	false alarm probability
P	sensing beam gain	$P_{D}$	1. -order Marcum Q form of false alarm probability
$R_{s}$	covariance of transmitted signal	$t\left(\phi_{r}\right)$	complex output of angular domain scanning

## Appendix A

This paper assumes that assumes that ideal channel state information can be obtained during the optimization process. When α=0, the optimization problem simplifies to:

(A1) $$\begin{array}{l} \underset{F,\Phi }{\max}\;f_{\mathrm{sen}}\left(F,\Phi \right)\\ s.t.\;\begin{array}{cc} \sum_{k=1}^{K}{\left\|f_{k}\right\|}_{2}^{2}\leq P_{T}, & \theta_{l}\in \left[0,2\pi \right],\forall l=1,2,\cdots ,L \end{array} \end{array}$$

where the sensing objective function takes the form $f_{\mathrm{sen}}\left(F,\Phi \right)={\left\|f_{k}\right\|}_{2}^{2}\cdot f\left(\Phi \right)$, and for any Φ, $f\left(\Phi \right)>0$ holds true for any possible RIS phase that may occur in practice. Under this assumption, regardless of the current channel implementation, any global optimal solution to problem Equation (A1) will satisfy ${\left\|{\hat{f}}_{k}\right\|}_{F}^{2}=P_{T}$.

Suppose $\left({\hat{f}}_{k},\hat{\Phi }\right)$ is an optimal solution to problem Equation (A1), but ${\left\|{\hat{f}}_{k}\right\|}_{F}^{2}<P_{T}$. Define ${\tilde{f}}_{k}=\sqrt{\frac{P_{T}}{{\left\|f_{k}^{*}\right\|}_{2}^{2}}}{\hat{f}}_{k}$, then ${\left\|{\tilde{f}}_{k}\right\|}_{2}^{2}=P_{T}$, and keep the beam direction unchanged. $f\left(\hat{\Phi }\right)$ remains constant, we have

(A2) $$f_{\mathrm{sen}}\left(\tilde{F},\hat{\Phi }\right)=P_{T}\cdot f\left(\Phi^{*}\right)>{\left\|f_{k}\right\|}_{2}^{2}\cdot f\left(\hat{\Phi }\right)=f_{\mathrm{sen}}\left({\hat{f}}_{k},\hat{\Phi }\right)$$

this contradicts optimality. Therefore, any optimal solution must have a power level that is saturated.

This conclusion does not depend on the specific form of the channel, but only on the linear relationship between the sensing gain and the transmission power. Even when accounting for the actual dynamics of the channel, as long as the BS obtains the instantaneous CSI for the coherent time block and solves Equation (A1). The optimization of F and the RIS phase shifts Φ remains highly dependent on the channel and must be solved iteratively using the algorithm proposed in this paper.

## Appendix B

The expanded expression of the objective function Equation (20) is the normalized weighted sum of the communication rate and the sensing beam gain.

(A3) $$f\left(\Phi \right)≜\frac{\alpha R}{R_{\max}}+\frac{\left(1-\alpha \right)P}{P_{\max}}$$

Define the auxiliary variables for gradient solution $V_{kq}=\left(h_{bu,q}+h_{ru,q}\phi_{p_{i}}h_{br,q}\right)f_{q}+\sigma_{w}^{2}$, $V_{ri}=b^{H}\left(\phi_{r}\right)\phi_{p_{i}}h_{br,i}$ and the derivative of the objective function with respect to RIS can be expressed as

(A4) $$\frac{\partial f}{\partial \phi_{l}}=\frac{\partial R}{\partial \phi_{l}}+\frac{\partial P}{\partial \phi_{l}}=\frac{{\left|V_{kq}\right|}^{2}\frac{\partial {\left|V_{kk}\right|}^{2}}{\partial \phi_{l}}-{\left|V_{kk}\right|}^{2}\frac{\partial {\left|V_{kq}\right|}^{2}}{\partial \phi_{l}}}{{\left|V_{kq}\right|}^{2}\left(1+\gamma_{k}\right)\ln2}+f_{k}\left(\frac{\partial {\left|V_{ri}\right|}^{2}}{\partial \phi_{l}}+2a_{t}^{H}\left(\phi_{r}\right)b^{H}\left(\phi_{r}\right)h_{br,l}\right)f_{k}^{H}$$

The total Euclidean gradient is expressed by Equation (21). At point $\phi_{l}\in M$, linearization of constraint $\left|\phi_{l}\right|=1$ gives $\phi_{l}^{*}d\phi_{l}+\phi_{l}d\phi_{l}^{*}=0$. For the Euclidean gradient vector $\frac{\partial f}{\partial \phi_{l}}$, decompose it into $\frac{\partial f}{\partial \phi_{l}}={\left(\frac{\partial f}{\partial \phi_{l}}\right)}_{T}+R\left(\frac{\partial f}{\partial \phi_{l}}⊙\phi_{l}^{*}\right)⊙\phi_{l}$. The overall cut space projection is expressed by Equation (22).

Using the standard Riemannian gradient descent, move along the gradient direction in the tangent space, and then contract the mapping back onto the manifold. For the constant modulus constraint, contraction normalization

(A5) $$\phi_{l}^{t+1}=\gamma_{p_{i}}\frac{\phi_{l}^{t}-\eta_{t}\nabla_{M}f\left(\phi_{l}\right)}{\left|\phi_{l}^{t}-\eta_{t}\nabla_{M}f\left(\phi_{l}\right)\right|}$$

where we will return the amplitude of the active elements to the phase shift coefficients based on the importance scoring matrix.

On the Riemannian manifold, the Newton directional solution of the linear system $\mathrm{Hess}\;f=-\nabla_{M}\;f$. Suppose there is a tangent vector ψ, which satisfies $R\left\{\psi ⊙\phi^{*}\right\}=0$ and there is an equivalent form $\psi ≜\frac{\partial f}{\partial \phi_{L}}$. Take the derivative along the direction ψ

(A6) $$\partial \left(\nabla f\left(\psi \right)\right)=\nabla^{2}f\left(\psi \right)-\nabla R\left\{\nabla f\left(\psi \right)⊙\psi^{*}\right\}⊙\psi$$

where $\nabla^{2}f$ represents the Euclidean Hessian. Project onto the tangent space. Since ψ is perpendicular to the tangent space, the term containing ⊙ψ is projected to zero. However, the vector is already in the tangent space, and the projection remains unchanged. For a complex manifold, the Riemann-Hessian can be expressed as

(A7) $$\mathrm{Hess}\;f\left(\Phi \right)\left[\psi \right]=\nabla^{2}f\left(\Phi \right)-R\left\{\nabla^{2}f\left(\Phi \right)⊙\Phi^{*}\right\}⊙\Phi -R\left\{\nabla f\left(\Phi \right)⊙\Phi^{*}\right\}⊙\psi$$

setting its gradient to zero to obtain the Newton Equation $\mathrm{Hess}\;f\left(\eta_{t}\right)=-\nabla f\left(\Phi_{t}\right)$. Regularize Newton’s Equations by adding a damping term, adaptive step size is Equation (26).

## Appendix C

The fixed F and $\omega_{k}$ are quadratic functions of $u_{k}$. Take the partial derivatives with respect to $u_{k}$ and set them to zero

(A8) $$\frac{\partial e_{k}}{\partial u_{k}^{*}}=u_{k}^{*}\left(\sum_{q}{\left|h_{b,k}f_{q}\right|}^{2}+\sigma_{w}^{2}\right)-h_{b,k}f_{k}=0$$

where we have obtained the optimal solution for the equalizer, Equation (11). Substitute $e_{k}$, and we obtain MMSE

(A9) $$e_{k}^{\mathrm{MMSE}}=1-\frac{{\left|h_{b,k}f_{k}\right|}^{2}}{\sum_{q=1}^{K}{\left|h_{b,k}f_{q}\right|}^{2}+\sigma_{w}^{2}}=\frac{1}{1+\gamma_{k}}$$

Fixed the value of $u_{k}$ as the optimal value. Take the derivative of $\log_{2}\omega_{k}-\frac{\omega_{k}e_{k}-1}{\ln2}$ with respect to w and set it to zero. The optimal value obtained from the objective function later will be R.

(A10) $$\frac{1}{\omega_{k}\ln2}-\frac{e_{k}^{\mathrm{MMSE}}}{\ln2}=0$$
